# *De Novo* Transcriptome Characterization of a Sterilizing Trematode Parasite (*Microphallus* sp.) from Two Species of New Zealand Snails

**DOI:** 10.1534/g3.116.037275

**Published:** 2017-01-23

**Authors:** Laura Bankers, Maurine Neiman

**Affiliations:** Department of Biology, University of Iowa, Iowa City, Iowa 52242

**Keywords:** transcriptome, host-parasite interactions, microsatellites, coevolution, trematode

## Abstract

Snail-borne trematodes represent a large, diverse, and evolutionarily, ecologically, and medically important group of parasites, often imposing strong selection on their hosts and causing host morbidity and mortality. Even so, there are very few genomic and transcriptomic resources available for this important animal group. We help to fill this gap by providing transcriptome resources from trematode metacercariae infecting two congeneric snail species, *Potamopyrgus antipodarum* and *P. estuarinus*. This genus of New Zealand snails has gained prominence in large part through the development of *P. antipodarum* and its sterilizing trematode parasite *Microphallus livelyi* into a textbook model for host–parasite coevolutionary interactions in nature. By contrast, the interactions between *Microphallus* trematodes and *P. estuarinus*, an estuary-inhabiting species closely related to the freshwater *P. antipodarum*, are relatively unstudied. Here, we provide the first annotated transcriptome assemblies from *Microphallus* isolated from *P. antipodarum* and *P. estuarinus*. We also use these transcriptomes to produce genomic resources that will be broadly useful to those interested in host–parasite coevolution, local adaption, and molecular evolution and phylogenetics of this and other snail–trematode systems. Analyses of the two *Microphallus* transcriptomes revealed that the two trematode types are more genetically differentiated from one another than are the *M. livelyi* infecting different populations of *P. antipodarum*, suggesting that the *Microphallus* infecting *P. estuarinus* represent a distinct lineage. We also provide a promising set of candidate genes likely involved in parasitic infection and response to salinity stress.

Snail-borne trematodes are important sources of morbidity and mortality in animals, including humans (Hotez 2013; Jurberg and Brindley 2015). For example, infections by trematodes in the genus *Schistosoma* affect >200 million people in sub-Saharan Africa (Rollinson *et al.* 2013). Trematodes and their mollusk intermediate hosts are also important models for the study of host–parasite coevolutionary interactions (*e.g.*, Lively and Jokela 1996; King *et al.* 2009). While digenetic trematodes (parasitic flatworms) are one of the largest groups of metazoan parasites, with ∼18,000 species (Cribb *et al.* 2001), the genomic and transcriptomic resources needed to understand trematode evolution, host–parasite coevolution, and the genomic basis of trematode infection are only available for 13 of these species (>50% members of the *Schistosoma* genus; NCBI, January 2017). Further development of such resources for this medically and scientifically important group of parasites is a necessary component of characterizing the molecular and genomic underpinnings of parasitic infection and host–parasite coevolutionary interactions.

New Zealand snails in the genus *Potamopyrgus* are often the intermediate host of sterilizing trematode parasites in the genus *Microphallus* (Winterbourn 1973). Here, we focus on *Microphallus* ‘*livelyi*’ (Hechinger 2012; hereafter “*M. livelyi*”), a species of digenetic trematode that uses *Potamopyrgus antipodarum* as an intermediate host and dabbling ducks (Anatinae) as the final host (Dybdahl and Lively 1996; Lively and Jokela 1996; King *et al.* 2009; Osnas and Lively 2011). Dabbling ducks are infected by *M. livelyi* while foraging, typically consuming infected *P. antipodarum* from submerged rocks and aquatic vegetation. The trematodes lay sexually produced eggs within the ducks, which are excreted within duck feces (Osnas and Lively 2011). The next round of snail infection begins after detritus-feeding *P. antipodarum* consume *M. livelyi* eggs while foraging along lake bottoms and in aquatic vegetation (Lively and Jokela 1996). Following snail consumption, the *M. livelyi* eggs clonally develop into thousands of encysted larvae (metacercariae) that rapidly sterilize the snail (Winterbourn 1973; King *et al.* 2009). *M. livelyi* individuals that cannot infect the first snail by which they are ingested will die before completing their lifecycle, and successful infection means that the *M. livelyi* metacercariae sterilize the snail, leading to strong reciprocal antagonistic selection pressures between these two species (King *et al.* 2011). These metacercariae are also the *M. lively* stage that is transmissible to the duck final host (Levri and Lively 1996) and thus represent an especially important point of no return in the life history of *Microphallus*: if the snail-inhabiting metacercariae are not ingested by the duck final host, the parasite will be unable to complete its life cycle (Lively and Jokela 1996; King *et al.* 2011). The importance of this metacercariae life stage is emphasized by new RNA sequencing (RNA-Seq) evidence that the presence of *M. livelyi* metacercariae influences patterns of gene expression in *P. antipodarum* (Bankers *et al.* 2016). By this logic and informed by these data, we chose to focus on the metacercariae stage for our analyses.

How interactions between *M. livelyi* and *P. antipodarum* play out in *Microphallus* are not well characterized, particularly at the genetic and molecular levels. The initial insights we do have into the mechanisms underlying *Microphallus* infectivity come from local adaptation and reciprocal infection experiments, providing preliminary lines of evidence in support of the likelihood that coevolutionary interactions between *P. antipodarum* and *M. livelyi* fit a “matching alleles” infection genetics model (Dybdahl and Krist 2004; Lively *et al.* 2004; King *et al.* 2009). With a matching alleles system, parasite infection success is thought to be determined by whether the parasite genotype at the loci involved in mediating infection match the corresponding loci in the host, enabling successful parasites to evade detection by host defenses. While matching alleles systems for infection have been demonstrated in several host–parasite interactions, the specific genetic or molecular mechanisms underlying such interactions remain unclear in *P. antipodarum* and in every other system (Carmona *et al.* 2015; Routtu and Ebert 2015).

The transcriptomic resources described here, the first genomic resources for the *Microphallus* genus of which we are aware, will provide a valuable starting point to characterize the molecular underpinnings of *M. livelyi* infection and the coevolutionary interactions between *M. livelyi* and *P. antipodarum*. We present annotated transcriptomes assemblies from *M. livelyi* metacercariae isolated from *P. antipodarum*, as well as from *Microphallus* of unknown species isolated from *P. estuarinus*, a closely related species native to New Zealand estuaries. While the coevolutionary interactions between *M. livelyi* and *P. antipodarum* are well characterized, much less is known about the *Microphallus* that infect *P. estuarinus* (Winterbourn 1973; Hechinger 2012). Expanding the genomic resources available for these two closely related types of *Microphallus* will allow for the characterization of genes that contribute broadly to infection processes, as well as genes that play species-specific roles in host–parasite interactions.

We identified and annotated 7584 pairs of one-to-one orthologous transcripts between the two transcriptomes, analyzed levels of genetic variation within and genetic differentiation between the two types of *Microphallus* at these genes, and performed gene ontology (GO)-based functional comparisons between these two samples. We also provide a broad set of genomic resources that set the stage for future studies aimed at characterizing the molecular underpinnings of the coevolutionary interactions between *Microphallus* and *Potamopyrgus*.

## Materials and Methods

### Sample collection and RNA-Seq

We isolated *M. livelyi* metacercariae from four individual adult female *P. antipodarum* (PA-*Microphallus*) collected in January of 2013 in shallow regions (<1 m lake depth) of each of the two New Zealand lakes Alexandrina and Kaniere, for a total of eight PA-*Microphallus* samples. We chose these lakes because their *P. antipodarum* populations contain relatively high frequencies of *M. livelyi* infection (10–20%; Vergara *et al.* 2013). We also isolated *Microphallus* metacercariae from a single adult female *P. estuarinus* (PE-*Microphallus*) collected from the Ashley River estuary (near Christchurch, New Zealand) in January of 2015. Metacercariae were collected from stage five infections (http://www.indiana.edu/∼curtweb/trematodes/DATA_KEY.HTM), in which the entire snail body cavity is filled with clonally produced metacercariae cysts. We chose the metacercariae life stage both because it represents a critical time point in the life cycle of *Microphallus* and because this life stage is easily identifiable and allows for larger volumes of tissue to be collected than earlier stages.

We dissected individual *Potamopyrgus* snails to determine infection status and saved parasite tissue from snails containing stage five *Microphallus* infections. We separated parasite from snail tissue with a forceps and used a micropipette to isolate metacercariae cysts. We immediately placed the metacercariae in RNAlater Solution (Life Technologies Corporation) at 4° for 24 hr, and subsequently stored the RNAlater-submerged metacercariae at −80° (according to the manufacturer’s protocol) until RNA extraction. The eight PA-*Microphallus* metacercariae samples were pooled in a single tube. We had to use this pooling strategy for the PA-*Microphallus* because at this time (early 2013), pooling of cysts from multiple snails was necessary to obtain a sufficient amount of RNA to perform RNA-Seq. Technological advances in library preparation and RNA-Seq between 2013 and 2015 meant that we were able to obtain a sufficient amount of RNA from the metacercariae isolated from a single *P. estuarinus* individual for RNA-Seq.

We extracted RNA from metacercariae following the TRIzol protocol (Chomczynski and Sacchi 1987; Chomczynski 1993). We assessed RNA quantity and quality using a Bio-Rad Experion Automated Electrophoresis Station and Experion RNA analysis kit, requiring an RQI ≥ 8 and minimum of 2 µg of total RNA (following the manufacturer’s protocol). We prepared cDNA libraries following the Illumina Truseq LS protocol (Illumina, San Diego, CA) for the PA-*Microphallus* and PE-*Microphallus* samples. We barcoded the PA-*Microphallus* library and pooled the barcoded library with 11 other barcoded samples that were part of a different project in one Illumina HiSeq 2000 lane in 2013. For the PE-*Microphallus* library, we barcoded the library and pooled the barcoded library with five other samples that were part of a different project in one Illumina HiSeq 2000 lane in 2015. We performed 2 × 100 bp paired-end RNA-Seq for both *Microphallus* libraries. We obtained 31,635,450 paired-end reads (mean read length = 101 bp) for PA-*Microphallus* and 4,16,15,863 paired-end reads for PE-*Microphallus* (mean read length = 126 bp).

### Transcriptome assembly and annotation

We used FASTQC (Andrews 2010) to assess RNA-Seq quality and the FASTX Toolkit (Gordon and Hannon 2010) to trim adapter sequences and remove poor quality reads from raw RNA-Seq data, requiring a mean Phred score ≥ 20. After quality filtering, 31,096,527 paired-end reads remained for PA-*Microphallus* and 40,413,790 paired-end reads remained for PE-*Microphallus*. We used these filtered reads to generate *de novo* transcriptome assemblies for each of the two *Microphallus* samples.

We performed the Trinity *in silico* normalization, requiring a minimum Kmer coverage of 2× and maximum Kmer coverage of 100× (following Haas *et al.* 2013), and otherwise default parameters (Grabherr *et al.* 2011). We then generated preliminary *de novo* transcriptome assemblies for both types of *Microphallus* using Trinity software with default parameters (Haas *et al.* 2013). Preliminary assemblies contained 65,665 contigs and 2,26,896 contigs for PA-*Microphallus* and PE-*Microphallus*, respectively. We used the Trinity plugin TransDecoder with default parameters to annotate likely protein-coding regions, identify long open reading frames of putative genes, and filter miscalled isoforms (Haas *et al.* 2013). Next, we used hierarchical clustering based on sequence identity to further reduce redundancy in the transcriptome assemblies, as implemented by CD-HIT-EST (Huang *et al.* 2010). To reduce the likelihood of collapsing truly nonredundant transcripts, we used a sequence identity of 0.95 and word size of 8 nt (*e.g.*, Li *et al.* 2002). These steps resulted in assemblies containing 15,435 contigs for PA-*Microphallus* and 29,564 contigs for PE-*Microphallus*. We mapped reads back to their respective filtered transcriptome assemblies using TopHat2 and then used Cufflinks (Trapnell *et al.* 2013) to estimate mean FPKM for each transcript in each transcriptome. Mean FPKM (±SD) for the transcriptome assemblies were 71.6 (±274.8) for PA-*Microphallus* and 46.8 (±284.3) for PE-*Microphallus*. The large SD in FPKM for both types of *Microphallus* is due to the large ranges in FPKM (∼0 to 10,866 for PA-*Microphallus*; ∼0 to 19,279 for PE-*Microphallus*). The nearly twofold higher number of transcripts for PE-*Microphallus* relative to PA-*Microphallus* was likely a combination of advances in sequencing chemistry and technology between 2013 and 2015, higher sequencing depth for PE-*Microphallus*, and/or differences in heterozygosity of the sequenced individuals and/or samples. We annotated the transcriptomes using blastx with an E-value cut-off of 1e−6, followed by Blast2GO (using default parameters) to assign GO terms to blastx-annotated transcripts (Conesa *et al.* 2005). For PA-*Microphallus*, we obtained both blastx and GO annotations for 9272 transcripts and only blastx annotations for an additional 3814 transcripts. For PE-*Microphallus* we obtained both blastx and GO annotations for 13,778 transcripts and only blastx annotations for an additional 8822 transcripts. This analysis revealed 143 transcripts involved in photosynthesis in the PE-*Microphallus* assembly (none in PA-*Microphallus*), representing potential contaminants in the snail gut from which the PE-*Microphallus* was isolated. These 143 transcripts were filtered out of subsequent analyses. Finally, we used BUSCO (Simão *et al.* 2015) to assess the completeness of our transcriptome assemblies. This program uses tblastn to identify conserved single-copy orthologs among groups of organisms. We used the BUSCO OrthoDB Metazoan database to search our assemblies for the presence, absence, duplication, and/or fragmentation of 843 conserved single-copy Metazoan orthologs.

### Identification of orthologous transcripts

We used “reciprocalblast_allsteps.py” (Warren *et al.* 2014) to identify putative orthologous transcripts between the two *Microphallus* transcriptomes based on one-to-one reciprocal best blastn hits. We performed reciprocal blastn searches between the two transcriptomes with an E-value cutoff of 1e−20 and identified pairs of transcripts that were reciprocally one another’s top blastn hit and that occurred once and only once in each transcriptome query. We identified 11,160 of such transcript pairs, which we will hereafter refer to as “one-to-one orthologs.” Using a custom python script (github.com/jsharbrough/grabContigs), we extracted the 11,160 orthologous sequences from each *Microphallus* transcriptome to generate putative ortholog transcriptomes for each sample. To improve the accuracy of our ortholog predictions, we then used blastx against the NCBI nonredundant dataset with an E-value cutoff of 1e−6 (Camacho *et al.* 2009) to annotate the two transcriptomes and filter out putative orthologs that received different blastx annotations. This step resulted in 7935 remaining putative one-to-one orthologs. To further ensure that we accurately predicted orthologs, we also performed reciprocal shortest molecular distance comparisons between the 7935 pairs of transcripts using “reciprocal_smallest_distance.py” (Wall *et al.* 2003). Here, we inferred orthologs using a combination of global sequence alignments between the two transcriptomes and maximum likelihood-based evolutionary pairwise distances, and identified orthologous pairs of transcripts as those sets of transcripts with the shortest pairwise molecular distance between them relative to their pairwise distances from any of the other 7934 transcripts in the dataset (Wall *et al.* 2003). This filtering step left 7584 remaining pairs of one-to-one orthologous transcripts that met all three of our criteria: (1) reciprocal best blastn hits, (2) the same blastx annotation, and (3) shared the shortest pairwise evolutionary distance relative to all other transcripts. There were 7851 and 21,980 transcripts for PA-*Microphallus* and PE-*Microphallus*, respectively, that did not meet these three criteria (hereafter, “nonone-to-one orthologs”). We extracted the 7584 orthologous transcripts from each reference transcriptome to generate ortholog transcriptomes for each of the two *Microphallus* datasets. We estimated mean FPKM for each transcript in the ortholog transcriptomes as described above. Mean FPKM (±SD) for the transcriptome assemblies were 114.3 (±403.2) for PA-*Microphallus* and 111.7 (±569.2) for PE-*Microphallus*.

### Comparisons of genetic variation and genetic differentiation

We used Genome Tools to split both of the ortholog transcriptomes into individual fasta files for each transcript (Gremme *et al.* 2013). We then merged pairs of orthologous sequences into multifasta files and used Clustal Omega to align and compute Jukes–Cantor-corrected pairwise molecular distances for each pair of orthologous transcripts (Sievers *et al.* 2011). Finally, we used the pairwise molecular distance values between each pair of orthologous transcripts obtained from Clustal Omega to estimate mean and median pairwise molecular distance between the two transcriptomes. We used SPSS Statistics v.23 to generate a boxplot of pairwise molecular distance that enabled us to visualize the distribution of these values (see Supplemental Material, Figure S1).

We used the PoPoolation pipeline to assess allelic diversity (Watterson’s θ) within and relative levels of genetic differentiation (*F_ST_*) between the two *Microphallus* samples (Kofler *et al.* 2011a,b). Watterson’s θ is an estimator of nucleotide diversity based on the number of segregating sites in a sample (here, SNPs) (Watterson 1975). *F_ST_* is a descriptive statistic that estimates relative levels of genetic differentiation among populations based on variance in allele frequencies (Holsinger and Weir 2009). Analyses of levels of nucleotide variation and genetic differentiation from pooled RNA-Seq data with programs like PoPoolation and PoPoolation2 (Kofler *et al.* 2011a,b), which were developed to perform variant calling and calculate *F_ST_* from pooled sequencing data, can effectively identity SNPs and candidate genes and perform *F_ST_*-based measures of genetic differentiation (*e.g.*, Fischer *et al.* 2013; Konczal *et al.* 2014; Li *et al.* 2014; Thavamanikumar *et al.* 2014; Ellison *et al.* 2016). In order to lower the amount of memory needed for analysis and to reduce biases linked to variation in gene expression among loci, we required a minimum coverage of 10× and a maximum coverage of 200× for both the θ and *F_ST_* analyses described below. We also required that each SNP was sequenced at least four times and filtered out SNPs with minor allele frequencies <5% (*e.g.*, Fischer *et al.* 2013; Konczal *et al.* 2014).

First, we used TopHat2 (Trapnell *et al.* 2013) to map *Microphallus* reads to their respective ortholog transcriptomes. For the Watterson’s θ estimates, this process included mapping the PA-*Microphallus* reads to the PA-*Microphallus* ortholog transcriptome and mapping the PE-*Microphallus* reads to the PE-*Microphallus* ortholog transcriptome. To estimate *F_ST_* between the two types of *Microphallus*, PoPoolation2 requires the mapping of the two samples (PA-*Microphallus* and PE-*Microphallus*) to a single reference. In order to account for the ortholog transcriptome to which reads were mapped, we performed this analysis twice, first by mapping both sets of *Microphallus* reads to the PA-*Microphallus* ortholog transcriptome, and second by mapping both sets of *Microphallus* reads to the PE-*Microphallus* ortholog transcriptome. After mapping, we used Picard Tools v.1.119 (http://broadinstitute.github.io/picard/) to process and filter the bam files. This step included the addition of read groups and removal of duplicate reads from each bam file. We then used GATK (McKenna *et al.* 2010; DePristo *et al.* 2011; Van der Auwera *et al.* 2013) to realign reads around indels and to reassign mapping quality. Finally, we used SAMtools (Li *et al.* 2012) to sort each bam file by coordinates relative to the ortholog transcriptome to which they were mapped and to generate mpileups.

We generated separate mpileup files for each bam file and used PoPoolation (Kofler *et al.* 2011a) for further analyses. PoPoolation is a suite of programs designed to handle pooled sequencing data and is able to account for the number of individuals sequenced in each pool. First, we used PoPoolation’s Variation-at-position.pl script (which is not available in newer program versions) to calculate Watterson’s θ for each orthologous transcript for the two *Microphallus* types. We compared the medians and distributions of θ for each transcriptome using Bonferroni-corrected Mann–Whitney *U* tests and Kolmogorov–Smirnov tests, respectively, as implemented in SPSS Statistics v.23. We also used SPSS Statistics v.23 to generate a boxplot for Watterson’s θ and *F_ST_* per SNP in order to visualize the distribution of values for each statistic (see Figure S1).

We then used the most up-to-date version of PoPoolation (PoPoolation2) (Kofler *et al.* 2011b) to generate synchronized mpileups (mpileup2sync.pl) for each bam file associated with the ortholog transcriptomes (*i.e.*, PA-*Microphallus* and PE-*Microphallus* mapped to the PA-*Microphallus* ortholog transcriptome, and PA-*Microphallus* and PE-*Microphallus* mapped to the PE-*Microphallus* ortholog transcriptome). Next, we used the PoPoolation2 fst-sliding.pl to calculate *F_ST_* per SNP to assess levels of relative genetic differentiation between the two *Microphallus* types. We used these *F_ST_* values to determine mean and median *F_ST_* per SNP between PA-*Microphallus* and PE-*Microphallus* relative to both of the ortholog transcriptomes. We applied outlier analyses within SPSS Statistics v.23 to identify transcripts containing *F_ST_* outlier SNPs (*i.e.*, SNPs with *F_ST_* values outside of the 95% C.I. around the mean), which can be interpreted as representing significant genetic differentiation for these genes between these two types of *Microphallus*. Finally, we used blastx and Blast2GO to annotate *F_ST_* outlier-containing transcripts and determine putative functions.

### GO and KEGG-based functional comparisons of transcriptomes and orthologs

We used blastx (Camacho *et al.* 2009) and Blast2GO (Conesa *et al.* 2005) to annotate the orthologous sequences for both *Microphallus* types. We also annotated the transcripts from each transcriptome that did not fall into orthologous gene sets (nonone-to-one orthologs). We then performed GO functional enrichment analyses, using Fisher’s Exact tests as implemented by Blast2GO (Conesa *et al.* 2005), to identify overrepresented *vs.* underrepresented functional groups in the set of orthologous transcripts relative to the rest of the transcriptome, and for the set of nonone-to-one orthologous transcripts relative to the rest of the transcriptome. All functional enrichment analyses were performed on both *Microphallus* samples. This analysis approach allowed us to compare overrepresented *vs.* underrepresented functional groups for orthologous and nonorthologous transcripts between the two types of *Microphallus* in order to identify which functional groups were expressed in both *Microphallus* samples *vs.* those functional groups that were uniquely enriched in each *Microphallus* type. We also predicted KEGG (Kyoto Encyclopedia of Genes and Genomes database) pathways for each transcriptome as implemented by Blast2GO. This analysis allowed us to (1) identify potential among-gene interactions, (2) identify sets of interacting genes of relevance to the *Microphallus* life cycle stage that we sequenced, and (3) assess whether different pathways are being expressed between the two samples. Finally, we used Blast+ to generate local BLAST databases for both annotated transcriptomes.

### Microsatellite loci and primer prediction

We used PrimerPro v.1.0 (https://webdocs.cs.ualberta.ca/∼yifeng/primerpro/) to identify potential microsatellite loci in the two *Microphallus* transcriptomes. We limited these analyses to the ortholog transcriptomes to ensure the transcripts in which we identified microsatellites were distinct loci and to allow future direct comparisons among different types of *Microphallus*. The PrimerPro v.1.0 pipeline first predicts microsatellite loci by identifying simple sequence repeats (SSRs) using MISA (MIcroSAtellite Identification Tool; http://pgrc.ipk-gatersleben.de/misa/misa.html) and then uses Primer3 (Untergasser *et al.* 2012) and BLAST (Camacho *et al.* 2009) to generate primers isolating these loci. After these steps, PrimerPro v.1.0 outputs the putative microsatellites, the primer sequences, their transcriptomic locations, and the primer properties. Following Qiu *et al.* (2016), we allowed for a minimum of 10 repeats for mononucleotide repeats, six for dinucleotide repeats, five for trinucleotide repeats, four for tetranucleotide repeats, three for pentanucleotide repeats, and three for hexanucleotide repeats, and identified compound microsatellites as instances where there were more than one microsatellite separated by ≤150 nt.

### Data availability

The raw RNA-Seq reads are deposited at NCBI Sequence Read Archive under the accessions SRR5170514 and SRR5170515 for PA-*Microphallus* and PE-*Microphallus*, respectively. The Transcriptome Shotgun Assembly projects have been deposited at DDBJ/EMBL/GenBank under the accessions GFFK00000000 and GFFL00000000, for PA-*Microphallus* and PE-*Microphallus*, respectively. The versions described in this paper are the first versions, GFFK01000000 and GFFL01000000. BLAST databases for each transcriptome are available at http://bioweb.biology.uiowa.edu/neiman/Potamomics.php. SNP and molecular distance data, microsatellite loci with predicted primer details, and transcriptome annotations are deposited at Dryad (doi: 10.5061/dryad.3ft30). Transcriptome annotation statistics and annotation details (see Table S1 and Table S4), a list of orthologous transcript IDs (see Table S2), boxplots to visualize distributions of θ, *F_ST_*, and pairwise distances (see Figure S1), GO functional enrichment and KEGG analyses of orthologous transcripts (see Table S6 and Table S7), annotations of *F_ST_* outlier-containing transcripts (see Table S3 and Table S5), and information about predicted microsatellite loci (see Figure S2, Table S8, and Table S9) are provided in the online supplemental information. The custom python program is available at github.com/jsharbrough/grabContigs. 

## Results and Discussion

### Transcriptome assembly and annotation

We generated *de novo* transcriptome assemblies for stage five *Microphallus* metacercariae isolated from field-collected PA-*Microphallus* and PE-*Microphallus*. Our final transcriptome assemblies contained 15,435 and 29,564 transcripts for PA-*Microphallus* and PE-*Microphallus*, respectively. The assembly for PE-*Microphallus* contained approximately twice as many transcripts and was 33% longer than the assembly for PA-*Microphallus*. The PA-*Microphallus* assembly had, on average, 22% longer transcripts and 23% larger N50 than the PE-*Microphallus* assembly (Table 1). The marked differences in these assembly statistics are, at least in part, likely linked to differences in sequencing depth and technological advances between the two rounds of sequencing but could also reflect, for example, biological differences between *Microphallus* found in freshwater (inhabiting *P. antipodarum*) *vs.* estuarine (inhabiting *P. estuarinus*) habitats and/or differences in genetic variation (*e.g.*, heterozygosity) of the sequenced samples.

**Table 1:** Assembly statistics for whole transcriptome assemblies and ortholog transcriptome assemblies for PA-*Microphallus* and PE-*Microphallus* (present study) as well as for two previously published Platyhelminthes transcriptomes: the trematode *Schistosoma mansoni* and cestode *Echinococcus granulosus*

Assembly Statistic	Whole Transcriptome		Ortholog Transcriptome		Previously Published Transcriptomes	
	PA-*Microphallus*	PE-*Microphallus*	PA-*Microphallus*	PE-*Microphallus*	*S. mansoni*	*E. granulosus*
No. of Transcripts	15,435	29,564	7584	7584	6122	1850
Min. Transcript Length	297	297	297	297	21	19
Max. Transcript Length	10,398	13,584	9285	12,381	933	787
Median Transcript Length	837	585	1173	1191	704	597
Mean Transcript Length (±SD)	1225.18 (1102.41)	959.67 (1007.52)	1505.35 (1157.72)	1565.81 (1287.64)	600.6	523.7
Transcriptome Length	1,89,10,587	2,83,71,675	1,14,16,539	1,18,75,112	36,76,790	9,68,901
N content (%)	0	0	0	0	2,548	0
GC (%)	47.25	48.05	47.22	47.21	NA	NA
N50	1782	1368	2019	2070	742	638
Reference	Present study	Present study	Present study	Present study	Protasio *et al.* (2012)	Tsai *et al.* (2013)

Both previously published transcriptomes were obtained from http://www.sanger.ac.uk/resources/downloads/helminths/.

We obtained both blastx and GO annotations for 60.1 and 46.6% of transcripts and only blastx annotations (including those with GO mapping, but not GO annotation) for an additional 24.6 and 29.8% of transcripts for PA-*Microphallus* and PE-*Microphallus*, respectively. There was a mean of ∼3.4 GO annotations per transcripts per transcriptome. The remaining 15.2% of PA-*Microphallus* transcripts and 23.6% of PE-*Microphallus* transcripts were unable to be annotated, which is typical for *de novo* assemblies of nonmodel taxa (*e.g.*, Guo *et al.* 2015; Theissinger *et al.* 2016). The species that were most frequently the top BLAST hit for a given transcript were most often other trematodes (10,053 transcripts, 77.4% for PA-*Microphallus*; 12,342 transcripts, 54.9% for PE-*Microphallus*), or nontrematode members of Platyhelminthes (an additional 332 transcripts, 2.6% for PA-*Microphallus*; 382 transcripts, 1.7% for PE-*Microphallus*) (see Table S1).

We used BUSCO to estimate the completeness of our transcriptome assemblies based on the presence, absence, duplication, and/or fragmentation of conserved Metazoan genes. Both transcriptomes contain complete transcripts for ∼68% of the 843 conserved single-copy Metazoan genes in the BUSCO database. Our transcriptome assemblies contain gene fragments for an additional 7.8% (PA-*Microphallus*) and 14.5% (PE-*Microphallus*) of the conserved single-copy Metazoan genes, for a total of 76.5% (PA-*Microphallus*) and 82.4% (PE-*Microphallus*) of the 843 BUSCO genes represented in the transcriptomes (Table 2). Considered together with our assembly statistics (Table 1), these results indicate that our transcriptomes are of good quality, are reasonably complete, and are qualitatively similar to other recently published *de novo* transcriptome assemblies (*e.g.*, Guo *et al.* 2015; Hara *et al.* 2015; Tassone *et al.* 2016; Theissinger *et al.* 2016), despite the differences in transcript number and N50 between the two transcriptome assemblies. Because we analyzed transcriptomes generated from only one stage of infection from a parasite with a complex life cycle, at least some of the missing BUSCO genes likely represent genes that are not expressed in metacercariae rather than due to sequencing or assembly limitations. Regardless, because stage five metacercariae are ready for transmission to the final host (Levri and Lively 1996), these data provide an excellent first step toward identifying the genes involved in a critical stage of parasite development.

**Table 2:** BUSCO assessment for transcriptome completeness

	Whole Transcriptome		Ortholog Transcriptome	
	PA-*Microphallus*	PE-*Microphallus*	PA-*Microphallus*	PE-*Microphallus*
No. of Complete Single-Copy Genes (%)	579 (68.7)	573 (68.0)	436 (51.7)	432 (51.3)
No. of Duplicated Genes (%)	0 (0)	2 (0.2)	0 (0)	0 (0)
No. of Fragmented Genes (%)	66 (7.8)	122 (14.5)	51 (6.1)	55 (6.5)
No. of Missing Genes (%)	198 (23.5)	146 (17.3)	356 (42.2)	356 (42.2)

Includes 843 conserved metazoan genes. Of these 843 genes, we assessed the number of genes that were complete, duplicated, fragmented, and missing among our reference transcriptomes and ortholog transcriptomes.

We generated local BLAST databases for both annotated *Microphallus* transcriptomes that allow for simple queries of nucleotide and protein sequences (http://bioweb.biology.uiowa.edu/neiman/Potamomics.php). This resource will be broadly useful for future studies geared toward identifying candidate genes from *Microphallus* as well as other related ecologically and/or medically important parasites by providing an easy way to search for genes in a well-characterized host–parasite system. These databases will also be an asset for phylogenetic or molecular evolution-focused studies that would benefit from the addition of data from Trematoda.

### Ortholog identification and levels of genetic variation within and genetic differentiation between Microphallus samples

We identified 7584 pairs of one-to-one orthologous transcripts between the two transcriptomes (see Table S2) and 7851 and 21,980 nonone-to-one orthologous transcripts for PA-*Microphallus* and PE-*Microphallus*, respectively. The two one-to-one ortholog transcriptomes contain higher mean transcript lengths (18.6% longer for PA-*Microphallus*; 38.7% longer for PE-*Microphallus*) and larger N50 values (11.7% greater N50 for PA-*Microphallus*; 33.9% greater N50 for PE-*Microphallus*) than their corresponding reference transcriptomes (Table 1). The longer transcripts and higher N50 values in the one-to-one ortholog sets relative to the reference transcriptomes indicate that the one-to-one orthologs represent a set of approximately full-length high quality genes that occur once and only once in both transcriptome assemblies. More than half (PA-*Microphallus*: 51.7%; PE-*Microphallus*: 51.2%) of the BUSCO genes are retained in this reduced gene set, further indicating that our filtering steps likely removed poor quality transcripts from our ortholog transcriptomes (Table 2). The annotation statistics for the ortholog transcriptomes are broadly similar to the annotation statistics for the reference transcriptomes (see Table S1), suggesting the ortholog transcriptomes are likely representative of the reference transcriptomes. We also obtained transcriptome assemblies for two previously published Platyhelminthes transcriptomes that were readily available, well-characterized, and among the top-hit species in our transcriptome annotations (http://www.sanger.ac.uk/resources/downloads/helminths/), the trematode *Schistosoma mansoni* (Protasio *et al.* 2012) and the cestode *Echinococcus granulosus* (Tsai *et al.* 2013). We used these transcriptomes to further assess the quality of our transcriptomes relative to previously published assemblies (Table 1 and Table S1); this comparison reinforces our conclusion that our transcriptomes are of high quality (*e.g.*, higher N50, mean transcript length).

Of the 7584 transcripts in the ortholog transcriptomes, a distinct majority received both blastx and GO annotation for PA-*Microphallus* (5746 transcripts, 75.8%) and PE-*Microphallus* (5793 transcripts, 76.4%). An additional 15.9% (1211 transcripts) and 15.8% (1203 transcripts) of these transcripts received only blastx annotations for PA-*Microphallus* and PE-*Microphallus*, respectively. The species that were top BLAST hits most often and the species that were hit most frequently in blastx searches include other trematodes (five species), as well as nontrematode Platyhelminthes (two species) and other metazoans with well-annotated genomic and/or transcriptomic resources (see Table S1). Species within Platyhelminthes were the top blastx hit for 90.6% (6337 transcripts) and 90.3% (6356 transcripts) of the orthologous transcripts that received blastx hits for PA-*Microphallus* and PE-*Microphallus*, respectively. The remaining 663 annotated orthologs for PA-*Microphallus* received top BLAST hits from mollusks (393 transcripts, 5.6%), other metazoans (253 transcripts, 3.6%), or nonmetazoan taxa (17 transcripts, 0.2%), and the remaining 682 annotated PE-*Microphallus* transcripts received top BLAST hits from mollusks (418 transcripts, 5.9%), other metazoans (250 transcripts, 3.6%), or nonmetazoan taxa (14 transcripts, 0.2%). The relatively small proportion of orthologous transcripts with a mollusk as their top hit could reflect low levels of contamination during the process of isolating metacercariae from snail tissue, but is likely also linked in part to availability of well-annotated genomic and/or transcriptomic resources for the specific species that were hit (see Table S1).

We used PoPoolation to calculate allelic diversity using SNPs (Watterson’s θ) for each orthologous transcript by mapping PA-*Microphallus* and PE-*Microphallus* reads to their own ortholog transcriptomes. Mean (±SD) θ per transcript for PA-*Microphallus* mapped to the PA-*Microphallus* orthologs was 0.0044 (±0.0039) and median θ was 0.0036. Mean (±SD) θ per transcript for PE-*Microphallus* mapped to the PE-*Microphallus* orthologs was 0.0051 (±0.0044) and median θ was 0.0046 (see Figure S1A). These estimates of θ are broadly consistent with observations from a previous allozyme-based estimate of θ (0.001–0.021) in several populations of *P. antipodarum*-infecting *Microphallus* (Dybdahl and Lively 1996). Comparisons of medians (Mann–Whitney *U* test) and distributions of θ (Kolmogorov–Smirnov test) for each transcriptome to one another revealed that θ values are significantly different between the two types of *Microphallus* (*P* < 0.001 for both analyses), with θ of PE-*Microphallus* being on average ∼0.9 times higher than PA-*Microphallus*. This result thus provides an initial line of evidence for higher genetic variation in PE-*Microphallus* relative to PA-*Microphallus*, though one obvious caveat to definitive interpretation of these results is the differences in sampling between the two types of *Microphallus* in our study. In particular, because the PA-*Microphallus* sample was produced from pooled RNA from multiple individuals, we cannot tie sequence differences back to individual worms. On the other hand, because we only sequenced a single PE-*Microphallus* individual, we can definitively ascribe within-sample variation to individual variation, but are not able to evaluate across-individual polymorphism.

Next, we used PoPoolation2 to estimate *F_ST_* at each SNP between PA-*Microphallus* and PE-*Microphallus*. This analysis allowed us to estimate relative levels of genetic differentiation among populations based on variance in allele frequencies between the two *Microphallus* samples (Holsinger and Weir 2009). In order to account for the ortholog transcriptome to which reads were mapped, we calculated *F_ST_* per SNP between PA-*Microphallus* and PE-*Microphallus* mapped to the PA-*Microphallus* ortholog transcriptome and calculated *F_ST_* per SNP between PA-*Microphallus* and PE-*Microphallus* mapped to the PE-*Microphallus* ortholog transcriptome. We found a mean (±SD) *F_ST_* per SNP between the two types of *Microphallus* mapped to the PA-*Microphallus* ortholog transcriptome of 0.129 (±0.129); median *F_ST_* per SNP was 0.085. Mean (±SD) *F_ST_* per SNP between the two types of *Microphallus* mapped to the PE-*Microphallus* ortholog transcriptome was 0.148 (±0.138), and median *F_ST_* per SNP was 0.102 (see Figure S1B). We supplemented these population genetic-based estimates of genetic differentiation with a phylogeny-based divergence estimation approach, using Clustal Omega to generate multiple sequence alignments and to calculate Jukes–Cantor-corrected pairwise molecular distance for each pair of orthologous transcripts. The mean pairwise molecular distance (±SD) between the two transcriptomes was 0.034 (±0.062) and the median was 0.019 (see Figure S1C).

These comparisons provide the first report of which we are aware suggesting that the *Microphallus* that infect *P. antipodarum* and *P. estuarinus* represent distinct genetic lineages. In particular, our *F_ST_*-based estimates of divergence between these two *Microphallus* types are an order of magnitude higher than the *F_ST_* values reported in an early allozyme-based study comparing *F_ST_* across populations of *Microphallus* infecting *P. antipodarum* (Dybdahl and Lively 1996). This result thus provides an initial line of evidence for marked genetic differentiation between these two *Microphallus* types relative to the across-population differentiation observed for *Microphallus* infecting *P. antipodarum*. Our pairwise molecular distance analyses further suggest that these two types of *Microphallus* are genetically distinct and are consistent with estimates of genetic variation from prior studies from across the genus. O’Dwyer *et al.* (2014) estimated pairwise molecular distance (uncorrected p-distance) at *cox1* to be ∼0.002 for lineages of *Microphallus* that were isolated from the gastropod mollusk *Austrolittorina cincta*, collected in Otago Harbor, New Zealand. By contrast, pairwise distances between these sequences and previously published *cox1* sequences of *Microphallus* infecting the mud snail *Zeacumantus subcarinatus* were orders of magnitude higher (0.21–0.25) than within the *A. cinta*-infecting *Microphallus*. Another relevant recent example comes from Galaktionov *et al.* (2012), who measured absolute pairwise differences and p-distances of ITS1 and ITS2 between six *Microphallus* species that infect gastropods from the genus *Littorina* from across the Holarctic, and found that pairwise distances within the *Microphallus* genus infecting congeneric gastropods (*Littorina*) ranged from 0 to 0.16 at these genes. Our results for mean pairwise molecular distance between PA-*Microphallus* and PE-*Microphallus* (0.034) are thus broadly consistent with other reports of variation within and among other *Microphallus* taxa. These new data also permit some useful comparisons: these pairwise distances are an order of magnitude higher than pairwise distances between *Microphallus* lineages collected from a single molluscan species (O’Dwyer *et al.* 2014), an order of magnitude lower than pairwise distances between *Microphallus* lineages collected from different genera of mollusks (O’Dwyer *et al.* 2014), and are within the range of previously reported pairwise distances for *Microphallus* lineages infecting congeneric gastropods (Galaktionov *et al.* 2012). Taken together, the outcomes of these analyses are consistent with a scenario where PA-*Microphallus* and PE-*Microphallus* represent distinct genetic lineages relative to *Microphallus* collected from different populations of *P. antipodarum*.

### Orthologous transcripts containing F_ST_ outlier SNPs

Using the *F_ST_* analyses described above, we identified 36 *F_ST_* outlier-containing transcripts between PA-*Microphallus* and PE-*Microphallus* when mapping to the PA-*Microphallus* ortholog transcriptome. Of these 36 transcripts, 31 received blastx and GO annotation, one received only blastx annotation, and four were unable to be annotated (see Table S3). A total of 31 of the annotated transcripts had top blastx hit species within Trematoda (see Table S4). Based on Blast2GO annotations, these *F_ST_* outlier-containing transcripts appear to be involved in cellular processes, metabolism, biological regulation, and localization (see Table S5). When mapping to the PE-*Microphallus* ortholog transcriptome, we identified 30 *F_ST_* outlier-containing transcripts. Of these transcripts, 22 received both blastx and GO annotations, three received only blastx annotation, and three were unable to be annotated (see Table S3). All 25 of the annotated transcripts received top blastx hit species within Platyhelminthes, 24 of which were within Trematoda (see Table S4). The annotated *F_ST_* outlier-containing transcripts identified when mapping to the PE-*Microphallus* orthologs appear to be involved in similar biological processes as those identified by mapping to PA*-Microphallus* (see Table S5).

Only two of the transcripts were identified as *F_ST_* outliers in both comparisons. Both of these transcripts could represent genes that are evolving relatively rapidly compared to their respective ortholog transcriptome and/or may be potential targets of selection in one or both *Microphallus* types. These candidate genes are *N*-α-acetyltransferase, which is involved in the *N*-terminal acetyltransferase C (NatC) complex and acetylation of amino acids, and permease 1 heavy chain, which is involved in maltose metabolic processes (see Table S3). Genes involved in the NatC pathway have been implicated in modulating stress tolerance in *Caenorhabditis elegans* (Warnhoff and Kornfeld 2015). In schistosomes (Trematoda), permease 1 heavy chain is characterized as playing an important role in nutrient uptake from hosts and is expressed during all major schistosome life stages (Krautz-Peterson *et al.* 2007). This information from a related trematode group with a broadly similar life cycle indicates that permease 1 heavy chain might also play a role in nutrient acquisition in *Microphallus*. More directed study of patterns of molecular evolution and the roles that these two genes play in response to physiological stresses associated with freshwater *vs.* estuarine environments (*N*-α-acetyltransferase), as well as nutrient uptake by parasites from hosts (permease 1 heavy chain), in *Microphallus* will provide more definitive insights into their functions.

### GO functional enrichment and KEGG pathway identification

We used Blast2GO to perform functional enrichment analyses comparing our ortholog transcriptomes against their associated reference transcriptomes, with the goal of identifying overrepresented *vs.* underrepresented functional groups that are shared between the two types of *Microphallus*. We also used functional enrichment analyses of our nonone-to-one orthologs against their associated reference transcriptomes to assess the types of genes that are overrepresented *vs.* underrepresented among the transcripts that did not represent one-to-one orthologs between PA-*Microphallus* and PE-*Microphallus*. These analyses revealed seven GO terms that were significantly underrepresented among orthologous transcripts in PA-*Microphallus* relative to the PA-*Microphallus* reference transcriptome. These same seven functional groups are significantly overrepresented among the nonone-to-one orthologs for PA-*Microphallus* relative to the PA-*Microphallus* reference transcriptome (see Table S6). For the PE-*Microphallus* orthologs relative to the PE-*Microphallus* reference, we identified 20 significantly underrepresented and six significantly overrepresented functional groups. There were five underrepresented and 14 overrepresented functional groups among the nonone-to-one PE-*Microphallus* orthologs relative to the PE-*Microphallus* transcriptome. All but four of the functional groups among the PE-*Microphallus* transcripts were only significantly overrepresented or underrepresented among either the one-to-one or nonone-to-one orthologs but were never significantly overrepresented or underrepresented in both transcript sets (see Table S6).

There were only two GO categories that were that were overrepresented or underrepresented among orthologs from both types of *Microphallus*. First, genes with functions related to hydrolase activity are underrepresented among one-to-one orthologs and overrepresented among nonone-to-one orthologs for both PA-*Microphallus* and PE-*Microphallus*. The presence of genes involved in hydrolase activity among genes that were significantly functionally enriched is interesting in light of evidence that hydrolases are involved in damaging epithelial lining of the digestive tracts of hosts by the parasitic nematode *Contracaeum rudolphii* (Dziekońska-Rynko and Rokicki 2005). Second, genes involved in carbohydrate metabolic processes are underrepresented among one-to-one orthologs for both types of *Microphallus* relative to the reference transcriptomes. Evidence that schistosome parasites dynamically shift carbohydrate metabolic processes throughout the course of their life cycle (Horemans *et al.* 1992) hint that these genes could also be of relevance to *Microphallus*. Altogether, both of these functional categories of genes provide a promising set of candidates for assessment of the changes in gene expression and physiological responses associated with the infection process in *Microphallus*. The marked differences in functional groups that are significantly overrepresented *vs.* underrepresented in the ortholog transcriptomes relative to the reference transcriptomes between the two *Microphallus* types suggest that the types of genes present in the two reference transcriptomes are often different. Whether this result is more a consequence of biological differences between PA-*Microphallus* and PE-*Microphallus* or differences in sequencing depth cannot be determined at this time but warrants future study.

Finally, we performed KEGG analyses as implemented in Blast2GO to identify the types of enzyme pathways that were being expressed in each transcriptome. This analysis identified 123 KEGG pathways in the PA-*Microphallus* transcriptome and 128 KEGG pathways in the PE-*Microphallus* transcriptome (see Table S7). All but 11 of the KEGG pathways that we identified were expressed in both transcriptomes, three were expressed in PA-*Microphallus* only, and eight were expressed in PE-*Microphallus* only. Most of the 120 pathways present in both *Microphallus* transcriptomes are involved in various metabolic processes (49 pathways, 40.2%) or biosynthetic processes (41 pathways, 33.6%). Of the 11 pathways only identified in a single transcriptome, the three pathways identified in only PA-*Microphallus* include the riboflavin metabolic pathway, the penicillin and cephalosporin biosynthesis pathway, and the biosynthesis of siderophore group nonribosomal peptides. The eight pathways that were identified in only PE-*Microphallus* include a variety of biosynthesis and metabolic processes (see Table S7). One such pathway, glycosphingolipid biosynthesis, has been implicated in response to salinity stress in *Litopenaeus vannamei*, the Pacific white shrimp (Chen *et al.* 2015). This result is suggestive of a scenario where these genes contribute to the ability of PE-*Microphallus* to tolerate salinity fluctuations associated with estuarine habitats. This important habitat difference between PA-*Microphallus* and PE-*Microphallus* is likely to differentially influence the selective pressures experienced by these two parasites and thus has the potential to influence patterns and rates of molecular evolution (*e.g.*, Mitterboeck *et al.* 2016), making genes involved in salinity tolerance and/or stress particularly interesting candidates for future study.

### Predictions of microsatellite loci and primers

We used MISA software to identify SSRs within the ortholog transcriptomes and used Primer3 and BLAST to predict primers that could be used for future microsatellite-based analyses of *Microphallus*. We identified 109 SSRs in 100 transcripts for PA-*Microphallus* and 117 SSRs in 107 transcripts for PE-*Microphallus*. We identified all six types of microsatellites for which we searched, with trinucleotide sequences with at least five repeats representing the most common microsatellite type (62.3% of SSRs identified in PA-*Microphallus* and 60.7% of SSRs identified in PE-*Microphallus*) (Table 3; see Figure S2). Eight and four SSRs, respectively, were defined as compound for PA-*Microphallus* and PE-*Microphallus* (Table 3). We also used perl scripts available with MISA (http://pgrc.ipk-gatersleben.de/misa/) to compile specific details about the primer sets (*e.g.*, location, size of product, and melting temperature). We generated predicted primers for 84 of the PA-*Microphallus* SSRs from 100 sequences and for 83 of the PE-*Microphallus* SSRs from 113 sequences (see Table S8 and Table S9).

**Table 3:** Summary of potential microsatellite loci for the PA-*Microphallus* and PE-*Microphallus* one-to-one ortholog transcriptome assemblies

	PA-*Microphallus*	PE-*Microphallus*
Total No. of Sequences Examined	7584	7584
Total Size of Examined Sequences (bp)	48,68,704	49,22,150
Total No. of Identified SSRs	109	117
No. of SSR-Containing Sequences	100	107
No. of Sequences Containing >1 SSR	9	9
No. of SSRs Present in Compound Formation	8	4
No. of Mononucleotide SSRs (≥10 repeats)	4	5
No. of Dinucleotide SSRs (≥6 repeats)	5	11
No. of Trinucleotide Repeats (≥5 repeats)	68	71
No. of Tetranucleotide Repeats (≥4 repeats)	6	7
No. of Pentanucleotide Repeats (≥3 repeats)	8	12
No. of Hexanucleotide Repeats (≥3 repeats)	18	11

Future studies will be able to use these markers to characterize, for example, population genetic structure within and genetic differentiation between these two types of *Microphallus* isolated from two ecologically distinct snail taxa. Comparing patterns of molecular evolution among these orthologous genes from more *Microphallus* individuals isolated from *P. antipodarum* and *P. estuarinus* will allow for research directed at characterizing the strength and efficacy of selection and the importance of population structure in the context of a prominent model for host–parasite interactions and coevolution. Researchers can also use these resources to perform cost-effective analyses incorporating many *Microphallus* individuals from multiple populations to address key questions regarding the evolutionary connections between these two types of *Microphallus* (*e.g.*, whether there is gene flow between the *Microphallus* infecting *P. antipodarum* and *P. estuarinus* and whether there is similar evidence for local adaptation and coevolution between *P. estuarinus* and *Microphallus* as exists for *P. antipodarum* and *M. livelyi*).

### Caveats and conclusions

Multiple recent studies have shown that pooled RNA-Seq data can be used to effectively identify SNPs and candidate genes and to perform *F_ST_*-based measures of genetic differentiation and other population genetic measures (*e.g.*, Fischer *et al.* 2013; Konczal *et al.* 2014; Li *et al.* 2014; Thavamanikumar *et al.* 2014; Ellison *et al.* 2016). We used analysis approaches [PoPoolation (used to estimate θ), and PoPoolation2 (used to estimate *F_ST_*)] that are designed to handle pooled sequencing data and explicitly incorporate number of individuals in the sequencing pools as a parameter in estimations of nucleotide variation and genetic differentiation (Kofler *et al.* 2011a,b). For these reasons, we believe that we applied the most appropriate means available to analyze our data.

Even so, it is important to acknowledge the potential caveats and limitations associated with pooled RNA-Seq approaches (Konczal *et al.* 2014). First, in order to reduce the likelihood of including artefactual SNPs, we required that each SNP to be sequenced at least four times and filtered out SNPs with minor allele frequencies <5%, at the risk of missing alleles at low frequencies. Second, differences in gene expression among individuals and/or loci can complicate comparisons among samples. We tried to minimize the effects of these potential expression differences by requiring a minimum of 10× coverage and a maximum of 200× coverage for both the θ and *F_ST_* estimates. Another concern is that allele frequency estimates may be less accurate in regions of low expression (Konczal *et al.* 2014). As such, requiring a minimum of 10× coverage should reduce this effect, but does generate potential to miss some lowly expressed SNPs. The existence of allele-specific expression may be problematic in leading to underestimates of the number of heterozygous sites and overestimates of *F_ST_* in pooled sequencing data. These concerns are at least partially obviated by results of simulations presented in Konczal *et al.* (2014), who found that allele-specific expression only influences allele frequency estimation in a small fraction (∼1%) of genes (in a data set).

In conclusion, our study provides a set of widely useful transcriptomic and genomic resources for an evolutionarily and ecologically important group of parasites. *P. antipodarum* and *M. livelyi* are a textbook system for the study of host–parasite coevolutionary interactions, and the data generated herein represent the first large-scale genome-based resources for *Microphallus*. The annotated transcriptome assemblies, orthologous gene sets, BLAST databases, SNPs, and microsatellite loci and primers will facilitate research for scientists interested in host–parasite coevolution, local adaption, and/or patterns of molecular evolution and phylogenetics of these and other trematode parasites. Our *F_ST_*, functional enrichment, and KEGG pathway analyses provide a promising set of candidate genes and pathways for future study and further characterization for potential roles in physiological and stress responses relevant to organisms inhabiting freshwater *vs.* estuarine environments, as well as genes involved in parasitic infection of host taxa.

## Supplementary Material

Supplemental material is available online at www.g3journal.org/lookup/suppl/doi:10.1534/g3.116.037275/-/DC1.
