# Supplementary figures and images for: De Novo Transcriptome Characterization of a Sterilizing Trematode Parasite (Microphallus sp.) from Two Species of New Zealand Snails
Source: G3 (Bethesda). 2017 Jan 23;7(3):871–80. doi: 10.1534/g3.116.037275 (PMC5345718; doi:10.1534/g3.116.037275)

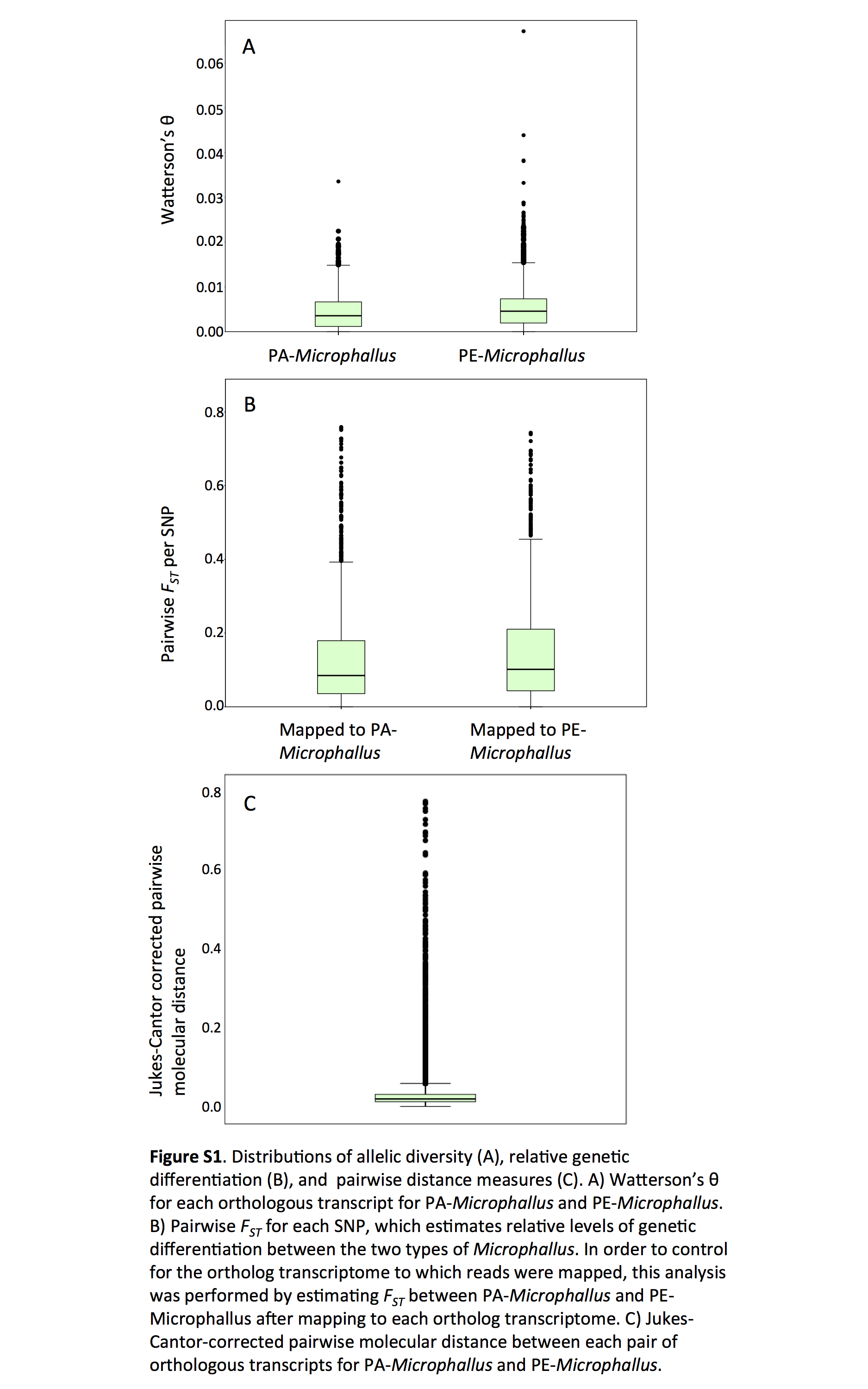

Supplement: Supplementary file 1 [file 871FigureS1.jpg]

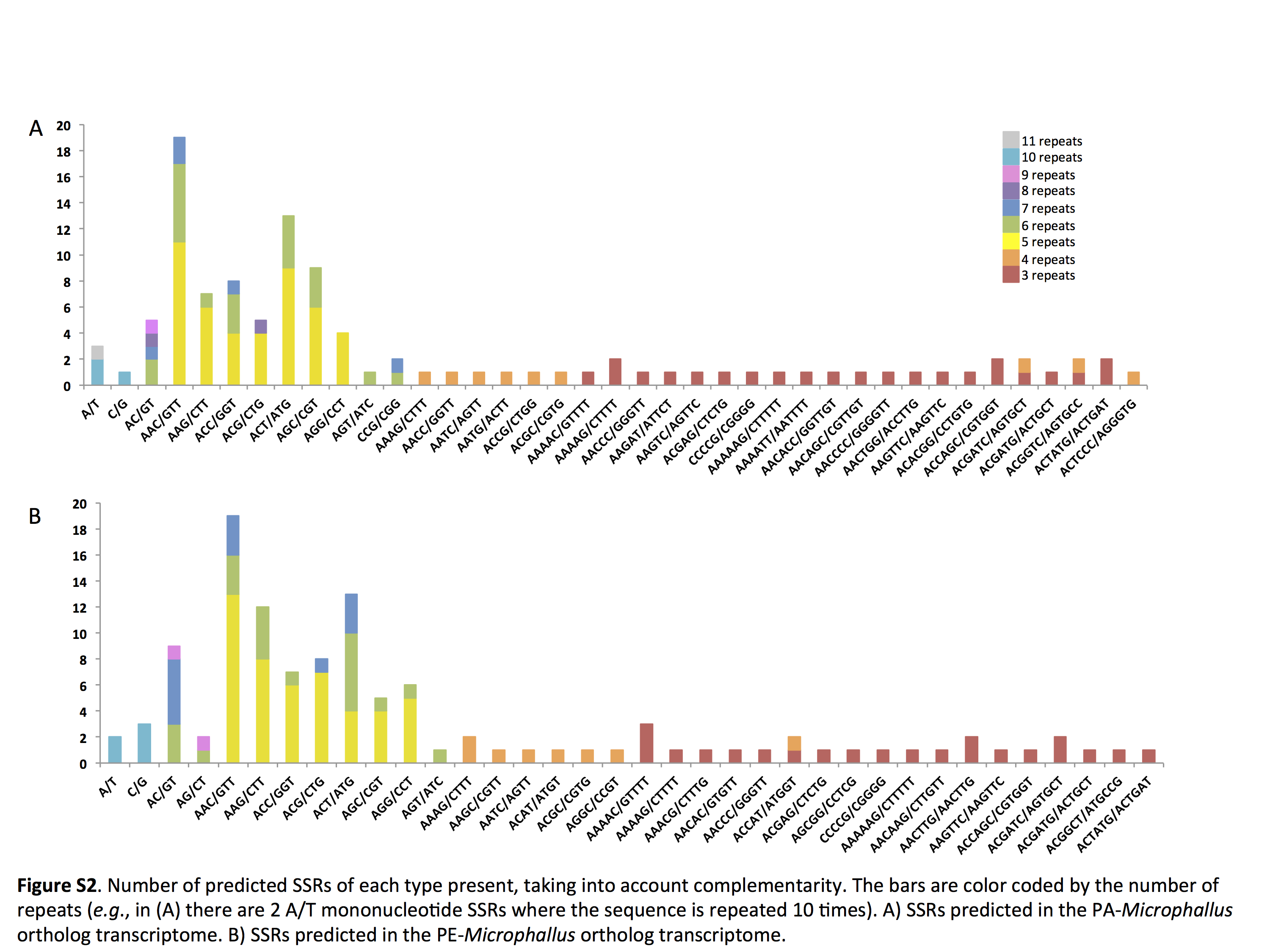

Supplement: Supplementary file 2 [file 871FigureS2.jpg]
