# Supplementary material for: De Novo Transcriptome Characterization of a Sterilizing Trematode Parasite (Microphallus sp.) from Two Species of New Zealand Snails
Source: G3 (Bethesda). 2017 Jan 23;7(3):871–80. doi: 10.1534/g3.116.037275 (PMC5345718; doi:10.1534/g3.116.037275)
Supplement: Supplementary file 3 [file 871TableS1.docx]

| **Table S1**. Annotation summary statistics for the PA-*Microphallus* and PE-*Microphallus* reference transcriptome assemblies and one-to-one-ortholog assemblies. "Rank-ordered top 10 top-hit species hit in blastx" refers to the 10 taxa that were most frequently the top blast hit for each transcriptome. "Rank-ordered top 10 species hit in blastx" refers to the 10 taxa that were hit the most frequently for each transcriptome. Green indicates Trematode, blue indicates non-trematode Platyhelminthes, orange indicates Mollusca, white indicates other. | | | | |
| --- | --- | --- | --- | --- |
|  | Whole transcriptome | | Ortholog transcriptome | |
| Annotation Statistic | PA-*Microphallus* | PE-*Microphallus* | PA-*Microphallus* | PE-*Microphallus* |
| # of transcripts with only blastx annotations (no GO mapping or annotation) | 2304 | 3321 | 1177 | 1170 |
| # of transcripts with blastx annotation and GO mapping (no GO annotation) | 1510 | 5501 | 34 | 33 |
| # of transcripts with both blastx and GO annotations | 9272 | 13778 | 5746 | 5793 |
| # of unannotated transcripts (no blastx or GO annotation) | 2349 | 6964 | 627 | 588 |
| Total # of GO annotations in assembly | 49313 | 92226 | 26037 | 26028 |
| Mean # of GO annotations per transcript (+/- SD) | 3.2 (5.8) | 3.1 (4.4) | 3.4 (4.5) | 3.4 (4.1) |
| Rank-ordered top 10 top-hit species hit in blastx (# of blast top hits) | *Clonorchis sinensis* (4195) | *Clonorchis sinensis* (5038) | *Opisthorchis viverrini* (2570) | *Opisthorchis viverrini* (2571) |
|  | *Opisthorchis viverrini* (3935) | *Opisthorchis viverrini* (4877) | *Clonorchis sinensis* (2504) | *Clonorchis sinensis* (2550) |
|  | *Schistosoma mansoni* (1169) | *Aplysia californica* (2342) | *Schistosoma mansoni* (503) | *Schistosoma mansoni* (502) |
|  | *Schistosoma japonicum* (737) | *Lottia gigantea* (1665) | *Schistosoma japonicum* (348) | *Schistosoma japonicum* (347) |
|  | *Aplysia californica* (373) | *Schistosoma mansoni* (1423) | *Schistosoma haematobium* (282) | *Schistosoma haematobium* (263) |
|  | *Lottia gigantea* (331) | *Crassostrea gigas* (1040) | *Aplysia californica* (91) | *Aplysia californica* (102) |
|  | *Crassostrea gigas* (263) | *Schistosoma japonicum* (943) | *Crassostrea gigas* (78) | *Crassostrea gigas* (83) |
|  | *Echinococcus granulosus* (191) | *Branchiostoma floridae* (240) | *Biomphalaria glabrata* (77) | *Lottia gigantea* (77) |
|  | *Hymenolepis microstoma* (141) | *Echinococcus granulosa* (222) | *Lottia gigantea* (65) | *Biomphalaria glabrata* (65) |
|  | *Saccoglossus kowalevskii* (52) | *Capitella teleta* (199) | *Echinococcus granulosus* (47) | *Echinococcus granulosus* (41) |
| Rank-ordered top-10 species hit in blastx (# of transcripts hit) | *Clonorchis sinensis* (13774) | *Clonorchis sinensis* (17057) | *Clonorchis sinensis* (6471) | *Clonorchis sinensis* (6512) |
|  | *Echinococcus granulosus* (12886) | *Echinococcus granulosa* (15230) | *Echinococcus granulosa* (6441) | *Echinococcus granulosa* (6489) |
|  | *Opisthorchis viverrini* (12234) | *Opisthorchis viverrini* (15106) | *Opisthorchis viverrini* (6162) | *Opisthorchis viverrini* (6190) |
|  | *Schistosoma mansoni* (11608) | *Schistosoma mansoni* (14608) | *Schistosoma mansoni* (5958) | *Schistosoma mansoni* (5974) |
|  | *Schistosoma japonicum* (9834) | *Schistosoma japonicum* (12852) | *Schistosoma japonicum* (4981) | *Schistosoma japonicum* (4918) |
|  | *Hymenolepis microstoma* (6702) | *Aplysia californica* (11857) | *Schistosoma haematobium* (4485) | *Schistosoma haematobium* (4525) |
|  | *Crassostrea gigas* (4926) | *Crassostrea gigas* (11624) | *Echinococcus multilocularis* (3795) | *Echinococcus multilocularis* (3820) |
|  | *Aplysia californica* (4875) | *Lottia gigantea* (10207) | *Hymenolepis microstoma* (3358) | *Hymenolepis microstoma* (3371) |
|  | *Lottia gigantea* (4599) | *Hymenolepis microstoma* (7987) | *Crassostrea gigas* (2033) | *Crassostrea gigas* (2087) |
|  | *Capitella teleta* (3421) | *Capitella teleta* (6716) | *Lingula anatina* (1608) | *Lingula anatina* (1679) |
