# Supplementary material for: De Novo Transcriptome Characterization of a Sterilizing Trematode Parasite (Microphallus sp.) from Two Species of New Zealand Snails
Source: G3 (Bethesda). 2017 Jan 23;7(3):871–80. doi: 10.1534/g3.116.037275 (PMC5345718; doi:10.1534/g3.116.037275)
Supplement: Supplementary file 5 [file 871TableS3.docx]

| **Table S3**. Blast2GO functional annotation of *F_ST_* outlier-containing transcripts, ranked by per-SNP *F_ST_.* | | | |
| --- | --- | --- | --- |
| PA-*Microphallus* and PE-*Microphallus* reads mapped to the PA-*Microphallus* ortholog transcriptome | | | |
| Transcript ID | Annotation | | per-SNP *F_ST_* |
| cds.comp12921_c0_seq1\|m.3539 | ---NA--- | | 0.75877 |
| cds.comp17286_c0_seq1\|m.14039 | ---NA--- | | 0.75500 |
| cds.comp19859_c0_seq1\|m.25348 | Y+L amino acid transporter 2 | | 0.75126 |
| cds.comp15683_c0_seq1\|m.8636 | Hypothetical protein CLF_100232 | | 0.72085 |
| cds.comp21073_c0_seq1\|m.31756 | Ubiquitin C-terminal hydrolase | | 0.71286 |
| cds.comp12494_c0_seq1\|m.3131 | Elongation factor 1-alpha | | 0.69820 |
| cds.comp17816_c0_seq1\|m.16083 | A kinase anchor 84 | | 0.66224 |
| cds.comp16608_c0_seq1\|m.11547 | Eukaryotic translation initiation factor subunit 7 zeta | | 0.64925 |
| cds.comp18117_c0_seq3\|m.17433 | Zinc finger 830 | | 0.64800 |
| cds.comp15755_c0_seq1\|m.8838 | Activator of 90 kDa heat shock ATPase homolog 1 | | 0.64727 |
| cds.comp17480_c1_seq1\|m.14734 | ---NA--- | | 0.63982 |
| cds.comp17257_c0_seq1\|m.13959 | N-acetyltransferase mak3 | | 0.63941 |
| cds.comp17991_c0_seq1\|m.16868 | PREDICTED: uncharacterized protein LOC105346399 isoform X2 | | 0.62901 |
| cds.comp18272_c0_seq1\|m.18112 | Cytochrome b5 reductase 1 | | 0.60812 |
| cds.comp14342_c0_seq1\|m.5600 | Ribosomal L18 | | 0.60526 |
| cds.comp18562_c0_seq3\|m.19409 | Phosphoglycerate mutase | | 0.57662 |
| cds.comp20247_c0_seq1\|m.27228 | Hypothetical protein T265_05124 | | 0.57382 |
| cds.comp16626_c0_seq1\|m.11589 | Trifunctional enzyme subunit mitochondrial | | 0.57247 |
| cds.comp20715_c0_seq1\|m.29871 | Innexin unc 9 | | 0.56643 |
| cds.comp20718_c0_seq1\|m.29881 | Leucyl aminopeptidase | | 0.56643 |
| cds.comp21016_c0_seq1\|m.31392 | Permease 1 heavy chain | | 0.53774 |
| cds.comp15314_c0_seq2\|m.7639 | Caspase apoptosis-related cysteine peptidase | | 0.53449 |
| cds.comp20265_c0_seq1\|m.27309 | RNA-binding Musashi homolog 2 | | 0.53249 |
| cds.comp17717_c0_seq1\|m.15616 | Der1 domain family | | 0.53013 |
| cds.comp20086_c0_seq2\|m.26421 | F-box WD repeat-containing partial | | 0.48715 |
| cds.comp18195_c0_seq1\|m.17744 | Poly(rC)-binding 3 | | 0.48636 |
| cds.comp21223_c0_seq1\|m.32593 | ---NA--- | | 0.48528 |
| cds.comp13200_c0_seq1\|m.3842 | BUD31 homolog | | 0.48210 |
| cds.comp16580_c0_seq1\|m.11475 | WD repeat-containing 92 | | 0.44564 |
| cds.comp16530_c0_seq1\|m.11357 | Nuclear migration nudC | | 0.44300 |
| cds.comp21175_c0_seq1\|m.32307 | T-cell immunomodulatory | | 0.44205 |
| cds.comp19425_c0_seq1\|m.23400 | 1,4-alpha-glucan-branching enzyme | | 0.44059 |
| cds.comp13509_c0_seq1\|m.4226 | Prefoldin subunit 3 | | 0.39826 |
| cds.comp15601_c0_seq1\|m.8402 | Rho-associated kinase 1 | | 0.39703 |
| cds.comp19826_c0_seq1\|m.25180 | Seryl tRNA Synthetase | | 0.39648 |
| cds.comp17620_c0_seq1\|m.15285 | Four and a half LIM domains 2 | | 0.39560 |
| PA-*Microphallus* and PE-*Microphallus* reads mapped to the PE-*Microphallus* ortholog transcriptome | | | |
| Transcript ID | | Annotation | per-SNP *F_ST_* |
| cds.comp112989_c1_seq4\|m.36825 | | Transmembrane 106A | 0.74337 |
| cds.comp108643_c0_seq1\|m.23357 | | Neuroendocrine 7b2 | 0.73953 |
| cds.comp114364_c0_seq2\|m.44261 | | Zinc transporter ZIP1 | 0.72085 |
| cds.comp109454_c0_seq1\|m.25052 | | cAMP-dependent kinase type II regulatory subunit | 0.69504 |
| cds.comp118020_c1_seq1\|m.75847 | | Myosin heavy chain | 0.69376 |
| cds.comp104096_c0_seq1\|m.17355 | | Hypothetical transcript | 0.68776 |
| cds.comp118673_c1_seq2\|m.82667 | | Permease 1 heavy chain | 0.67166 |
| cds.comp114713_c0_seq5\|m.46477 | | Solute carrier family 5 | 0.66851 |
| cds.comp107652_c0_seq1\|m.21674 | | N-alpha-acetyltransferase 30 isoform X1 | 0.65637 |
| cds.comp116947_c0_seq1\|m.64571 | | Tubulin polyglutamylase ttll6-like isoform X1 | 0.65634 |
| cds.comp109303_c0_seq1\|m.24725 | | Actin-related 2 3 complex subunit 2 | 0.63581 |
| cds.comp117143_c0_seq1\|m.66171 | | Mitochondrial uncoupling 4 | 0.61602 |
| cds.comp96623_c0_seq1\|m.12409 | | ---NA--- | 0.61337 |
| cds.comp108640_c0_seq1\|m.23344 | | ---NA--- | 0.61309 |
| cds.comp103307_c0_seq1\|m.16680 | | ---NA--- | 0.57564 |
| cds.comp96299_c0_seq1\|m.12227 | | DNA directed rna polymerases i ii and iii | 0.57508 |
| cds.comp104023_c0_seq1\|m.17264 | | Eukaryotic translation initiation factor 3 subunit 5 | 0.56347 |
| cds.comp96079_c0_seq1\|m.12141 | | 60S ribosome subunit biogenesis NIP7 | 0.54116 |
| cds.comp112555_c0_seq1\|m.35062 | | Cathepsin L | 0.53647 |
| cds.comp116061_c0_seq1\|m.56689 | | 26S proteasome non ATPase regulatory subunit | 0.53574 |
| cds.comp106626_c0_seq2\|m.20215 | | 5 -nucleotidase domain-containing 3 | 0.48304 |
| cds.comp112160_c0_seq1\|m.33233 | | Calcium-binding mitochondrial carrier S -3 | 0.48185 |
| cds.comp110523_c0_seq1\|m.27756 | | Hypothetical protein T265_06635 | 0.47938 |
| cds.comp112112_c0_seq11\|m.33043 | | V type proton ATPase 116 kDa subunit a | 0.47878 |
| cds.comp110683_c0_seq1\|m.28161 | | Epididymal secretory E1 precursor (Niemann Pick type C2 homolog) | 0.46730 |
| cds.comp105784_c0_seq1\|m.19114 | | N-acetyltransferase ARD1 homolog | 0.46657 |
| cds.comp114608_c7_seq1\|m.45874 | | Lipopolysaccharide induced TNF alpha factor | 0.46629 |
| cds.comp118739_c0_seq2\|m.83515 | | Family C2 unassigned peptidase (C02 family) | 0.46469 |
