# Supplementary material for: De Novo Transcriptome Characterization of a Sterilizing Trematode Parasite (Microphallus sp.) from Two Species of New Zealand Snails
Source: G3 (Bethesda). 2017 Jan 23;7(3):871–80. doi: 10.1534/g3.116.037275 (PMC5345718; doi:10.1534/g3.116.037275)
Supplement: Supplementary file 6 [file 871TableS4.docx]

| **Table S4**. The top ten top-hit species for the PA-*Microphallus* and PE-*Microphallus* ortholog transcriptome assemblies. Species are rank ordered by the number of transcripts with that species as the top blastx hit. Green indicates Trematoda, blue indicates non-trematode Platyhelminthes, orange indicates Mollusca. | |
| --- | --- |
| PA-*Microphallus* and PE-*Microphallus* reads mapped to the PA-*Microphallus* ortholog transcriptome | |
| Rank-ordered top 10 top-hit species hit in blastx | # top blastx hits |
| *Clonorchis sinensis* | 11 |
| *Opisthorchis viverrini* | 11 |
| *Schistosoma mansoni* | 6 |
| *Schistosoma haematobium* | 2 |
| *Aplysia californica* | 1 |
| *Schistosoma japonicum* | 1 |
| *Crassostrea gigas* | 1 |
| PA-*Microphallus* and PE-*Microphallus* reads mapped to the PE-*Microphallus* ortholog transcriptome | |
| Rank-ordered top 10 top-hit species hit in blastx | # top blastx hits |
| *Clonorchis sinensis* | 9 |
| *Opisthorchis viverrini* | 9 |
| *Schistosoma mansoni* | 2 |
| *Schistosoma japonicum* | 2 |
| *Echinococcus multilocularis* | 1 |
| *Schistosoma haematobium* | 1 |
| *Paragonimus westermani* | 1 |
