# Supplementary material for: De Novo Transcriptome Characterization of a Sterilizing Trematode Parasite (Microphallus sp.) from Two Species of New Zealand Snails
Source: G3 (Bethesda). 2017 Jan 23;7(3):871–80. doi: 10.1534/g3.116.037275 (PMC5345718; doi:10.1534/g3.116.037275)
Supplement: Supplementary file 7 [file 871TableS5.docx]

| **Table S5**. Level two gene ontology (GO) terms for *F_ST_* outlier-containing transcripts ordered alphabetically by GO term. The three major GO categories are represented by BP (Biological Processes), MF (Molecular Function), and CC (Cellular Components). | | | | | |
| --- | --- | --- | --- | --- | --- |
| PA-*Microphallus* and PE-*Microphallus* reads mapped to the PA-*Microphallus* ortholog transcriptome | | | | | |
| GO ID | GO term | GO Category | | # of transcripts | |
| GO:0005488 | binding | MF | | 18 | |
| GO:0065007 | biological regulation | BP | | 3 | |
| GO:0003824 | catalytic activity | MF | | 13 | |
| GO:0005623 | cell | CC | | 15 | |
| GO:0044464 | cell part | CC | | 15 | |
| GO:0071840 | cellular component organization or biogenesis | BP | | 3 | |
| GO:0009987 | cellular process | BP | | 18 | |
| GO:0051179 | localization | BP | | 2 | |
| GO:0032991 | macromolecular complex | CC | | 6 | |
| GO:0016020 | membrane | CC | | 9 | |
| GO:0044425 | membrane part | CC | | 9 | |
| GO:0008152 | metabolic process | BP | | 18 | |
| GO:0043226 | organelle | CC | | 4 | |
| GO:0044422 | organelle part | CC | | 2 | |
| GO:0050789 | regulation of biological process | BP | | 2 | |
| GO:0044699 | single-organism process | BP | | 9 | |
| PA-*Microphallus* and PE-*Microphallus* reads mapped to the PE-*Microphallus* ortholog transcriptome | | | | | |
| GO ID | GO term | | GO Category | | # of transcripts |
| GO:0005488 | binding | | MF | | 9 |
| GO:0065007 | biological regulation | | BP | | 6 |
| GO:0003824 | catalytic activity | | MF | | 12 |
| GO:0005623 | cell | | CC | | 14 |
| GO:0044464 | cell part | | CC | | 14 |
| GO:0071840 | cellular component organization or biogenesis | | BP | | 4 |
| GO:0009987 | cellular process | | BP | | 17 |
| GO:0051179 | localization | | BP | | 5 |
| GO:0032991 | macromolecular complex | | CC | | 9 |
| GO:0016020 | membrane | | CC | | 8 |
| GO:0044425 | membrane part | | CC | | 8 |
| GO:0008152 | metabolic process | | BP | | 13 |
| GO:0098772 | molecular function regulator | | MF | | 2 |
| GO:0043226 | organelle | | CC | | 10 |
| GO:0044422 | organelle part | | CC | | 5 |
| GO:0050789 | regulation of biological process | | BP | | 4 |
| GO:0044699 | single-organism process | | BP | | 8 |
| GO:0005215 | transporter activity | | MF | | 3 |
