# Supplementary material for: De Novo Transcriptome Characterization of a Sterilizing Trematode Parasite (Microphallus sp.) from Two Species of New Zealand Snails
Source: G3 (Bethesda). 2017 Jan 23;7(3):871–80. doi: 10.1534/g3.116.037275 (PMC5345718; doi:10.1534/g3.116.037275)
Supplement: Supplementary file 8 [file 871TableS6.docx]

| **Table S6**. Functionally enriched GO terms for PA-Microphallus and PE-Microphallus one-to-one ortholog transcriptomes and non-one-to-one ortholog transcriptomes relative to their respective reference assemblies. GO terms are ranked by *p*-value. "% in Test" refers to the percent of transcripts in the test assembly assigned a particular GO term. "% in Reference" refers to the percent of transcripts in the reference assembly assigned a particular GO term. Over/Under refers to whether a particular GO term was overrepresented vs. underrepresented relative to the reference transcriptome for that comparison. The three major GO categories are represented by BP (Biological Processes), MF (Molecular Function), and CC (Cellular Components). | | | | | | | | | | |
| --- | --- | --- | --- | --- | --- | --- | --- | --- | --- | --- |
| Functional enrichment analysis of orthologous transcripts from PA-*Microphallus* relative to the PA-*Microphallus* reference transcriptome, including whether each GO term is over vs. underrepresented in the test data set (ortholog transcriptome) relative to the reference transcriptome. | | | | | | | | | | |
| GO-ID | Term | Category | | FDR | | *p* | | % in Test | % in Reference | Over/Under |
| GO:0003777 | Microtubule motor activity | MF | | 1.10E-03 | | 3.21E-07 | | 0.81 | 2.06 | Under |
| GO:0001539 | Cilium or flagellum-dependent cell motility | BP | | 2.64E-03 | | 1.29E-06 | | 0.17 | 0.87 | Under |
| GO:0005858 | Axonemal dynein complex | CC | | 3.52E-03 | | 2.06E-06 | | 0.23 | 0.97 | Under |
| GO:0004553 | Hydrolase activity, hydrolyzing O-glycosyl compounds | MF | | 1.20E-02 | | 1.29E-05 | | 0.99 | 2.11 | Under |
| GO:0015074 | DNA integration | BP | | 1.20E-02 | | 1.38E-05 | | 0.60 | 1.53 | Under |
| GO:0005975 | Carbohydrate metabolic process | BP | | 1.20E-02 | | 1.41E-05 | | 3.01 | 4.76 | Under |
| GO:0005044 | Scavenger receptor activity | MF | | 1.46E-02 | | 1.99E-05 | | 0.10 | 0.61 | Under |
| Functional enrichment analysis of orthologous transcripts from PE-*Microphallus* relative to the PE-*Microphallus* reference transcriptome, including whether each GO term is over vs. underrepresented in the test data set (ortholog transcriptome) relative to the reference transcriptome. | | | | | | | | | | |
| GO-ID | Term | Category | FDR | | *p* | | % in Test | | % in Reference | Over/Under |
| GO:0005975 | Carbohydrate metabolic process | BP | 1.07E-05 | | 4.58E-07 | | 3.07 | | 4.93 | Under |
| GO:0006091 | Generation of precursor metabolites and energy | BP | 6.51E-05 | | 3.65E-06 | | 1.83 | | 3.21 | Under |
| GO:0003735 | Structural constituent of ribosome | MF | 4.06E-04 | | 2.61E-05 | | 2.21 | | 3.55 | Under |
| GO:0016491 | Oxidoreductase activity | MF | 4.74E-04 | | 3.28E-05 | | 4.60 | | 6.37 | Under |
| GO:0016798 | Hydrolase activity, acting on glycosyl bonds | MF | 7.99E-04 | | 6.06E-05 | | 1.17 | | 2.14 | Under |
| GO:0006520 | Cellular amino acid metabolic process | BP | 1.83E-03 | | 1.81E-04 | | 1.83 | | 2.91 | Under |
| GO:0019843 | rRNA binding | MF | 2.18E-03 | | 2.31E-04 | | 0.36 | | 0.92 | Under |
| GO:0009056 | Catabolic process | BP | 5.75E-03 | | 8.18E-04 | | 7.17 | | 8.87 | Under |
| GO:0006412 | Translation | BP | 5.75E-03 | | 9.05E-04 | | 4.19 | | 5.53 | Under |
| GO:0005840 | Ribosome | CC | 5.75E-03 | | 9.11E-04 | | 3.05 | | 4.23 | Under |
| GO:0043167 | Ion binding | MF | 6.16E-03 | | 9.97E-04 | | 23.17 | | 25.81 | Under |
| GO:0016023 | Cytoplasmic, membrane-bounded vesicle | CC | 8.30E-03 | | 1.51E-03 | | 2.76 | | 3.83 | Under |
| GO:0005783 | Endoplasmic reticulum | CC | 9.06E-03 | | 1.67E-03 | | 2.83 | | 3.90 | Under |
| GO:0005829 | Cytosol | CC | 1.17E-02 | | 2.19E-03 | | 7.17 | | 8.72 | Under |
| GO:0090304 | Nucleic acid metabolic process | BP | 1.47E-02 | | 2.81E-03 | | 8.45 | | 10.08 | Under |
| GO:0016301 | Kinase activity | MF | 2.00E-02 | | 3.96E-03 | | 7.10 | | 5.79 | Over |
| GO:0006629 | Lipid metabolic process | BP | 2.59E-02 | | 5.65E-03 | | 2.86 | | 3.79 | Under |
| GO:0034330 | Cell junction organization | BP | 2.86E-02 | | 6.62E-03 | | 0.79 | | 0.41 | Over |
| GO:0051186 | Cofactor metabolic process | BP | 3.27E-02 | | 7.80E-03 | | 1.60 | | 2.30 | Under |
| GO:0007267 | Cell-cell signaling | BP | 3.27E-02 | | 8.09E-03 | | 2.26 | | 1.60 | Over |
| GO:0048870 | Cell motility | BP | 3.27E-02 | | 8.11E-03 | | 2.71 | | 1.98 | Over |
| GO:0022857 | Transmembrane transporter activity | MF | 3.52E-02 | | 8.95E-03 | | 4.95 | | 3.96 | Over |
| GO:0006790 | Sulfur compound metabolic process | BP | 4.00E-02 | | 1.03E-02 | | 0.83 | | 1.35 | Under |
| GO:0005576 | Extracellular region | CC | 4.10E-02 | | 1.08E-02 | | 7.40 | | 8.71 | Under |
| GO:0004871 | Signal transducer activity | MF | 4.12E-02 | | 1.10E-02 | | 2.43 | | 1.75 | Over |
| GO:0042254 | Ribosome biogenesis | BP | 4.47E-02 | | 1.22E-02 | | 1.76 | | 2.44 | Under |
| Functional enrichment analysis of non-orthologous transcripts from PA-*Microphallus* relative to the PA-*Microphallus* reference transcriptome including, whether each GO term is over vs. underrepresented in the test data set (ortholog transcriptome) relative to the reference transcriptome. | | | | | | | | | | |
| GO-ID | Term | Category | FDR | | *p* | | % in Test | | % in Reference | Over/Under |
| GO:0003777 | Microtubule motor activity | MF | 1.10E-03 | | 3.21E-07 | | 2.06 | | 0.81 | Over |
| GO:0001539 | Cilium or flagellum-dependent cell motility | BP | 2.64E-03 | | 1.29E-06 | | 0.87 | | 0.17 | Over |
| GO:0005858 | Axonemal dynein complex | CC | 3.52E-03 | | 2.06E-06 | | 0.97 | | 0.23 | Over |
| GO:0004553 | Hydrolase activity, hydrolyzing O-glycosyl compounds | MF | 1.20E-02 | | 1.29E-05 | | 2.11 | | 0.99 | Over |
| GO:0015074 | DNA integration | BP | 1.20E-02 | | 1.38E-05 | | 1.53 | | 0.60 | Over |
| GO:0005975 | Carbohydrate metabolic process | BP | 1.20E-02 | | 1.41E-05 | | 4.76 | | 3.01 | Over |
| GO:0005044 | Scavenger receptor activity | MF | 1.46E-02 | | 1.99E-05 | | 0.61 | | 0.10 | Over |
| Functional enrichment analysis of non-orthologous transcripts from PE-*Microphallus* relative to the PE-*Microphallus* reference transcriptome including, whether each GO term is over vs. underrepresented in the test data set (ortholog transcriptome) relative to the reference transcriptome. | | | | | | | | | | |
| GO-ID | Term | Category | FDR | | *p* | | % in Test | | % in Reference | Over/Under |
| GO:0055114 | Oxidation-reduction process | BP | 2.74E-03 | | 8.59E-06 | | 5.58 | | 3.81 | Over |
| GO:1901575 | Organic substance catabolic process | BP | 3.02E-03 | | 9.66E-06 | | 8.04 | | 5.93 | Over |
| GO:0022626 | Cytosolic ribosome | CC | 3.50E-03 | | 1.15E-05 | | 1.61 | | 0.71 | Over |
| GO:0050662 | Coenzyme binding | MF | 4.71E-03 | | 1.78E-05 | | 1.73 | | 0.81 | Over |
| GO:0003735 | Structural constituent of ribosome | MF | 6.33E-03 | | 2.61E-05 | | 3.55 | | 2.21 | Over |
| GO:0016491 | Oxidoreductase activity | MF | 7.70E-03 | | 3.28E-05 | | 6.37 | | 4.60 | Over |
| GO:0019344 | Cysteine biosynthetic process | BP | 9.07E-03 | | 4.06E-05 | | 0.38 | | 0.02 | Over |
| GO:0017156 | Calcium ion regulated exocytosis | BP | 1.12E-02 | | 5.17E-05 | | 0.11 | | 0.50 | Under |
| GO:0006090 | Pyruvate metabolic process | BP | 1.20E-02 | | 5.71E-05 | | 1.02 | | 0.38 | Over |
| GO:0016798 | Hydrolase activity, acting on glycosyl bonds | MF | 1.25E-02 | | 6.06E-05 | | 2.14 | | 1.17 | Over |
| GO:0016051 | Carbohydrate biosynthetic process | BP | 1.33E-02 | | 6.51E-05 | | 1.23 | | 0.52 | Over |
| GO:0005216 | Ion channel activity | MF | 1.69E-02 | | 8.88E-05 | | 1.16 | | 2.05 | Under |
| GO:0007610 | Behavior | BP | 2.88E-02 | | 1.70E-04 | | 1.12 | | 1.95 | Under |
| GO:0015935 | Small ribosomal subunit | CC | 3.21E-02 | | 1.92E-04 | | 1.39 | | 0.67 | Over |
| GO:0072330 | Monocarboxylic acid biosynthetic process | BP | 3.70E-02 | | 2.26E-04 | | 0.61 | | 0.17 | Over |
| GO:0019843 | rRNA binding | MF | 3.73E-02 | | 2.31E-04 | | 0.92 | | 0.36 | Over |
| GO:0008021 | Synaptic vesicle | CC | 4.51E-02 | | 2.93E-04 | | 0.21 | | 0.62 | Under |
| GO:0043168 | Anion binding | MF | 4.51E-02 | | 2.95E-04 | | 14.29 | | 12.00 | Over |
| GO:0086001 | Cardiac muscle cell action potential | BP | 4.66E-02 | | 3.12E-04 | | 0.04 | | 0.29 | Under |
