# Supplementary material for: De Novo Transcriptome Characterization of a Sterilizing Trematode Parasite (Microphallus sp.) from Two Species of New Zealand Snails
Source: G3 (Bethesda). 2017 Jan 23;7(3):871–80. doi: 10.1534/g3.116.037275 (PMC5345718; doi:10.1534/g3.116.037275)
Supplement: Supplementary file 9 [file 871TableS7.docx]

| **Table S7.** Kyoto Encyclopedia of Genes and Genomes (KEGG) pathway analysis of the PA-*Microphallus* and PE-*Microphallus* reference transcriptome assemblies. KEGG pathways are ordered alphabetically. The "#of transcripts" refers to the number of transcripts known to be a part of a given pathway that were identified in the assembly. Pathways in gray indicate pathways present in one transcriptome assembly but not the other. | | | |
| --- | --- | --- | --- |
| PA-*Microphallus* | | PE-*Microphallus* | |
| KEGG pathway | # of transcripts | KEGG pathway | # of transcripts |
| Acarbose and validamycin biosynthesis | 1 | Acarbose and validamycin biosynthesis | 1 |
| Aflatoxin biosynthesis | 4 | Aflatoxin biosynthesis | 3 |
| Alanine, aspartate and glutamate metabolism | 20 | Alanine, aspartate and glutamate metabolism | 37 |
| Alpha-Linolenic acid metabolism | 13 | Alpha-Linolenic acid metabolism | 21 |
| Amino sugar and nucleotide sugar metabolism | 51 | Amino sugar and nucleotide sugar metabolism | 79 |
| Aminoacyl-tRNA biosynthesis | 46 | Aminoacyl-tRNA biosynthesis | 79 |
| Aminobenzoate degradation | 136 | Aminobenzoate degradation | 232 |
| Arachidonic acid metabolism | 12 | Arachidonic acid metabolism | 18 |
| Arginine and proline metabolism | 12 | Arginine and proline metabolism | 44 |
| Arginine biosynthesis | 17 | Arginine biosynthesis | 23 |
| Ascorbate and aldarate metabolism | 8 | Ascorbate and aldarate metabolism | 37 |
| Benzoate degradation | 12 | Benzoate degradation | 17 |
| Beta-Alanine metabolism | 16 | Beta-Alanine metabolism | 34 |
| Beta-Lactam resistance | 1 | Beta-Lactam resistance | 2 |
| Betalain biosynthesis | 2 | Betalain biosynthesis | 4 |
| Biosynthesis of ansamycins | 1 | Biosynthesis of ansamycins | 9 |
| Biosynthesis of antibiotics | 150 | Biosynthesis of antibiotics | 359 |
| Biosynthesis of siderophore group nonribosomal peptides | 1 | Biosynthesis of siderophore group nonribosomal peptides | 0 |
| Biosynthesis of unsaturated fatty acids | 9 | Biosynthesis of unsaturated fatty acids | 21 |
| Biosynthesis of vancomycin group antibiotics | 1 | Biosynthesis of vancomycin group antibiotics | 1 |
| Biotin metabolism | 3 | Biotin metabolism | 1 |
| Butanoate metabolism | 21 | Butanoate metabolism | 41 |
| Butirosin and neomycin biosynthesis | 4 | Butirosin and neomycin biosynthesis | 6 |
| C5-Branched dibasic acid metabolism | 4 | C5-Branched dibasic acid metabolism | 7 |
| Caffeine metabolism | 1 | Caffeine metabolism | 3 |
| Caprolactam degradation | 10 | Caprolactam degradation | 12 |
| Carbapenem biosynthesis | 0 | Carbapenem biosynthesis | 3 |
| Carbon fixation pathways in prokaryotes | 37 | Carbon fixation pathways in prokaryotes | 59 |
| Chloroalkane and chloroalkene degradation | 2 | Chloroalkane and chloroalkene degradation | 23 |
| Citrate cycle (TCA cycle) | 34 | Citrate cycle (TCA cycle) | 80 |
| Cutin, suberine and wax biosynthesis | 1 | Cutin, suberine and wax biosynthesis | 4 |
| Cyanoamino acid metabolism | 6 | Cyanoamino acid metabolism | 11 |
| Cysteine and methionine metabolism | 18 | Cysteine and methionine metabolism | 59 |
| D-Arginine and D-ornithine metabolism | 0 | D-Arginine and D-ornithine metabolism | 2 |
| D-Glutamine and D-glutamate metabolism | 2 | D-Glutamine and D-glutamate metabolism | 3 |
| Diterpenoid biosynthesis | 0 | Diterpenoid biosynthesis | 3 |
| Drug metabolism - cytochrome P450 | 18 | Drug metabolism - cytochrome P450 | 24 |
| Drug metabolism - other enzymes | 59 | Drug metabolism - other enzymes | 92 |
| Ether lipid metabolism | 7 | Ether lipid metabolism | 13 |
| Ethylbenzene degradation | 5 | Ethylbenzene degradation | 9 |
| Fatty acid biosynthesis | 7 | Fatty acid biosynthesis | 4 |
| Fatty acid degradation | 20 | Fatty acid degradation | 45 |
| Fatty acid elongation | 14 | Fatty acid elongation | 20 |
| Flavone and flavonol biosynthesis | 0 | Flavone and flavonol biosynthesis | 2 |
| Folate biosynthesis | 9 | Folate biosynthesis | 10 |
| Fructose and mannose metabolism | 28 | Fructose and mannose metabolism | 63 |
| Galactose metabolism | 19 | Galactose metabolism | 34 |
| Geraniol degradation | 12 | Geraniol degradation | 17 |
| Glucosinolate biosynthesis | 1 | Glucosinolate biosynthesis | 2 |
| Glutathione metabolism | 31 | Glutathione metabolism | 45 |
| Glycerolipid metabolism | 19 | Glycerolipid metabolism | 43 |
| Glycerophospholipid metabolism | 33 | Glycerophospholipid metabolism | 29 |
| Glycine, serine and threonine metabolism | 24 | Glycine, serine and threonine metabolism | 50 |
| Glycolysis / Gluconeogenesis | 41 | Glycolysis / Gluconeogenesis | 131 |
| Glycosaminoglycan biosynthesis - chondroitin sulfate / dermatan sulfate | 2 | Glycosaminoglycan biosynthesis - chondroitin sulfate / dermatan sulfate | 2 |
| Glycosaminoglycan biosynthesis - heparan sulfate / heparin | 3 | Glycosaminoglycan biosynthesis - heparan sulfate / heparin | 3 |
| Glycosaminoglycan biosynthesis - keratan sulfate | 1 | Glycosaminoglycan biosynthesis - keratan sulfate | 4 |
| Glycosaminoglycan degradation | 18 | Glycosaminoglycan degradation | 26 |
| Glycosphingolipid biosynthesis - ganglio series | 12 | Glycosphingolipid biosynthesis - ganglio series | 18 |
| Glycosphingolipid biosynthesis - globo series | 8 | Glycosphingolipid biosynthesis - globo series | 16 |
| Glycosphingolipid biosynthesis - lacto and neolacto series | 0 | Glycosphingolipid biosynthesis - lacto and neolacto series | 1 |
| Glycosylphosphatidylinositol(GPI)-anchor biosynthesis | 3 | Glycosylphosphatidylinositol(GPI)-anchor biosynthesis | 5 |
| Glyoxylate and dicarboxylate metabolism | 31 | Glyoxylate and dicarboxylate metabolism | 57 |
| Histidine metabolism | 10 | Histidine metabolism | 34 |
| Indole alkaloid biosynthesis | 4 | Indole alkaloid biosynthesis | 4 |
| Inositol phosphate metabolism | 26 | Inositol phosphate metabolism | 25 |
| Isoquinoline alkaloid biosynthesis | 10 | Isoquinoline alkaloid biosynthesis | 12 |
| Limonene and pinene degradation | 5 | Limonene and pinene degradation | 25 |
| Linoleic acid metabolism | 5 | Linoleic acid metabolism | 6 |
| Lipoic acid metabolism | 1 | Lipoic acid metabolism | 3 |
| Lysine biosynthesis | 0 | Lysine biosynthesis | 3 |
| Lysine degradation | 39 | Lysine degradation | 85 |
| Metabolism of xenobiotics by cytochrome P450 | 19 | Metabolism of xenobiotics by cytochrome P450 | 23 |
| Methane metabolism | 21 | Methane metabolism | 51 |
| Monobactam biosynthesis | 1 | Monobactam biosynthesis | 3 |
| mTOR signaling pathway | 17 | mTOR signaling pathway | 22 |
| Mucin type O-Glycan biosynthesis | 1 | Mucin type O-Glycan biosynthesis | 2 |
| N-Glycan biosynthesis | 18 | N-Glycan biosynthesis | 21 |
| Naphthalene degradation | 1 | Naphthalene degradation | 1 |
| Nicotinate and nicotinamide metabolism | 33 | Nicotinate and nicotinamide metabolism | 30 |
| Nitrogen metabolism | 22 | Nitrogen metabolism | 17 |
| Nitrotoluene degradation | 1 | Nitrotoluene degradation | 1 |
| Novobiocin biosynthesis | 1 | Novobiocin biosynthesis | 3 |
| One carbon pool by folate | 9 | One carbon pool by folate | 21 |
| Other glycan degradation | 33 | Other glycan degradation | 48 |
| Other types of O-glycan biosynthesis | 3 | Other types of O-glycan biosynthesis | 6 |
| Oxidative phosphorylation | 46 | Oxidative phosphorylation | 80 |
| Pantothenate and CoA biosynthesis | 8 | Pantothenate and CoA biosynthesis | 8 |
| Penicillin and cephalosporin biosynthesis | 1 | Penicillin and cephalosporin biosynthesis | 0 |
| Pentose and glucuronate interconversions | 13 | Pentose and glucuronate interconversions | 49 |
| Pentose phosphate pathway | 28 | Pentose phosphate pathway | 66 |
| Peptidoglycan biosynthesis | 2 | Peptidoglycan biosynthesis | 2 |
| Phenylalanine metabolism | 18 | Phenylalanine metabolism | 17 |
| Phenylalanine, tyrosine and tryptophan biosynthesis | 2 | Phenylalanine, tyrosine and tryptophan biosynthesis | 5 |
| Phenylpropanoid biosynthesis | 16 | Phenylpropanoid biosynthesis | 32 |
| Phosphatidylinositol signaling system | 48 | Phosphatidylinositol signaling system | 32 |
| Phosphonate and phosphinate metabolism | 4 | Phosphonate and phosphinate metabolism | 7 |
| Polyketide sugar unit biosynthesis | 1 | Polyketide sugar unit biosynthesis | 1 |
| Porphyrin and chlorophyll metabolism | 19 | Porphyrin and chlorophyll metabolism | 46 |
| Primary bile acid biosynthesis | 9 | Primary bile acid biosynthesis | 10 |
| Propanoate metabolism | 22 | Propanoate metabolism | 41 |
| Purine metabolism | 732 | Purine metabolism | 1250 |
| Pyrimidine metabolism | 101 | Pyrimidine metabolism | 170 |
| Pyruvate metabolism | 31 | Pyruvate metabolism | 85 |
| Retinol metabolism | 4 | Retinol metabolism | 17 |
| Riboflavin metabolism | 2 | Riboflavin metabolism | 0 |
| Selenocompound metabolism | 8 | Selenocompound metabolism | 16 |
| Sphingolipid metabolism | 19 | Sphingolipid metabolism | 48 |
| Starch and sucrose metabolism | 31 | Starch and sucrose metabolism | 76 |
| Steroid biosynthesis | 0 | Steroid biosynthesis | 4 |
| Steroid degradation | 3 | Steroid degradation | 1 |
| Steroid hormone biosynthesis | 4 | Steroid hormone biosynthesis | 10 |
| Streptomycin biosynthesis | 6 | Streptomycin biosynthesis | 15 |
| Styrene degradation | 1 | Styrene degradation | 3 |
| Sulfur metabolism | 3 | Sulfur metabolism | 8 |
| Synthesis and degradation of ketone bodies | 5 | Synthesis and degradation of ketone bodies | 14 |
| T cell receptor signaling pathway | 96 | T cell receptor signaling pathway | 170 |
| Taurine and hypotaurine metabolism | 6 | Taurine and hypotaurine metabolism | 6 |
| Terpenoid backbone biosynthesis | 12 | Terpenoid backbone biosynthesis | 16 |
| Tetracycline biosynthesis | 4 | Tetracycline biosynthesis | 3 |
| Thiamine metabolism | 605 | Thiamine metabolism | 1030 |
| Toluene degradation | 9 | Toluene degradation | 12 |
| Tropane, piperidine and pyridine alkaloid biosynthesis | 7 | Tropane, piperidine and pyridine alkaloid biosynthesis | 5 |
| Tryptophan metabolism | 22 | Tryptophan metabolism | 57 |
| Tyrosine metabolism | 15 | Tyrosine metabolism | 20 |
| Ubiquinone and other terpenoid-quinone biosynthesis | 2 | Ubiquinone and other terpenoid-quinone biosynthesis | 3 |
| Valine, leucine and isoleucine biosynthesis | 2 | Valine, leucine and isoleucine biosynthesis | 7 |
| Valine, leucine and isoleucine degradation | 29 | Valine, leucine and isoleucine degradation | 66 |
| Various types of N-glycan biosynthesis | 17 | Various types of N-glycan biosynthesis | 23 |
| Vitamin B6 metabolism | 3 | Vitamin B6 metabolism | 6 |
| Xylene degradation | 0 | Xylene degradation | 2 |
